# Increased hazard of death in community-tested cases of SARS-CoV-2 Variant of Concern 202012/01

**DOI:** 10.1101/2021.02.01.21250959

**Published:** 2021-02-03

**Authors:** Nicholas G. Davies, Christopher I. Jarvis, CMMID COVID-19 Working Group, W. John Edmunds, Nicholas P. Jewell, Karla Diaz-Ordaz, Ruth H. Keogh

**Affiliations:** 1. Centre for Mathematical Modelling of Infectious Diseases, London School of Hygiene and Tropical Medicine, London, UK.; 2. Department of Medical Statistics, Faculty of Epidemiology and Population Health, London School of Hygiene and Tropical Medicine, London, UK.; 3. Centre for Statistical Methodology, London School of Hygiene and Tropical Medicine, London, UK.

## Abstract

VOC 202012/01, a SARS-CoV-2 variant first detected in the United Kingdom in September 2020, has spread to multiple countries worldwide. Several studies have established that this novel variant is more transmissible than preexisting variants of SARS-CoV-2, but have not identified whether the new variant leads to any change in disease severity. We analyse a large database of SARS-CoV-2 community test results and COVID-19 deaths for England, representing approximately 47% of all SARS-CoV-2 community tests and 7% of COVID-19 deaths in England from 1 September 2020 to 22 January 2021. Fortuitously, these SARS-CoV-2 tests can identify VOC 202012/01 because mutations in this lineage prevent PCR amplification of the spike gene target (S gene target failure, SGTF). We estimate that the hazard of death among SGTF cases is 30% (95% CI 9–56%) higher than among non-SGTF cases after adjustment for age, sex, ethnicity, deprivation level, care home residence, local authority of residence and date of test. In absolute terms, this increased hazard of death corresponds to the risk of death for a male aged 55–69 increasing from 0.56% to 0.73% (95% CI 0.60–0.86%) over the 28 days following a positive SARS-CoV-2 test in the community. Correcting for misclassification of SGTF, we estimate a 35% (12–64%) higher hazard of death associated with VOC 202012/01. Our analysis suggests that VOC 202012/01 is not only more transmissible than preexisting SARS-CoV-2 variants but may also cause more severe illness.

SARS-CoV-2 Variant of Concern (VOC) 202012/01 (lineage B.1.1.7) carries several mutations, including a 6-nucleotide deletion that prevents amplification of the S gene target by a commercial PCR assay commonly used for community SARS-CoV-2 testing in England. By linking individual records of positive community tests with and without S gene target failure (SGTF) to COVID-19 deaths, we estimate the relative hazard of death from COVID-19 associated with infection by VOC 202012/01. We consider any PCR result with Ct < 30 for ORF1ab, Ct < 30 for N, and no detectable S (Ct > 40) to be SGTF; any PCR result with cycle threshold (Ct) < 30 for each of ORF1ab, N, and S to be non-SGTF; and exclude any other PCR result from the analysis.

## Characteristics of the study population

The study sample (Table 1) includes a total of 858,181 individuals who had a positive Pillar 2 (community) test between 1 November 2020 and 11 January 2021 processed by a laboratory capable of producing an SGTF reading. Of these, 48% had SGTF. Females comprised 52.4% of the sample; 45.3% were aged 1–34 years, 35.2% aged 35–54, 15.0% aged 55–69, 3.8% aged 70–85 and 0.7% aged 85 or older. The sex and age distributions were similar between those with SGTF and without. The SGTF group had a slightly smaller percentage of females and tended to be slightly younger (with 3.8% aged over 70 with SGTF versus 5.1% without SGTF). The majority of individuals lived in standard residential accommodation, with 0.5% living in a care or nursing home. Overall, 74.4% were White, 14.6% Asian, 3.9% Black and 7.0% of other, mixed or unknown ethnicity. The SGTF group had a slightly greater percentage of individuals in the Black and other, mixed or unknown ethnicity groups. More substantial differences were seen between the SGTF groups when looking at index of multiple deprivation (IMD) decile and NHS region. The SGTF group tended to have higher IMD (more deprived). The prevalence of SGTF was low until the end of November 2020, with 95% of the observed SGTF cases appearing after 29 November 2020.

**Fig. 1.**
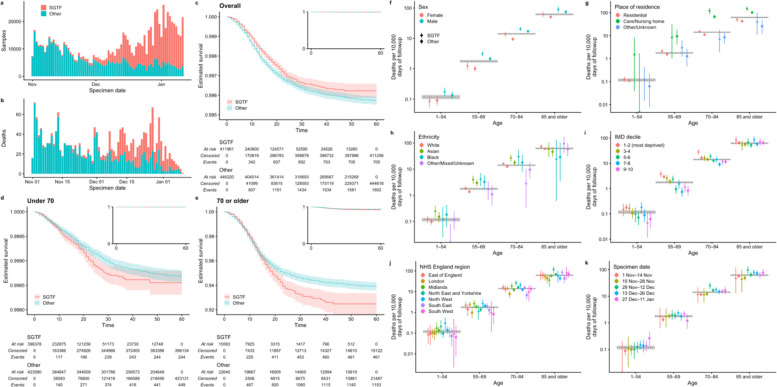
Descriptive analyses. **a** The number of samples with and without SGTF by day from 1 November 2020 to 11 January 2021. **b** Number of deaths within 28 days of positive test by specimen date included in the analysis. **c, d, e** Kaplan-Meier plots showing survival in individuals tested in the community in England with SGTF versus without in (**c**) all individuals, (**d**) individuals under 70, and (**e**) individuals 70 years or older. The apparent better survival in the SGTF group in the whole study cohort (**c**) is due to confounding by age. Insets show the full *y*–axis range. **f–k** Crude death rates (with 95% confidence intervals) in SGTF versus other for deaths within 28 days of positive test stratified by broad age groups and (**f**) sex, (**g**) place of residence, (**h**) ethnicity, (**i**) index of multiple deprivation, (**j**) NHS England region, and (**k**) specimen date. Grey ribbons show the overall crude death rates by age group irrespective of SGTF status.

The highest prevalences of SGTF over the study period were observed in the East of England (71.9%), South East (71.8%) and London (71.0%) NHS England regions. Prevalence of SGTF was lowest in the North East and Yorkshire region. SGTF prevalence was similar in males and females but lower in the older age groups: 48.6% in the 1–34 year olds compared with 36.3% in those aged 85 and older. In keeping with these age patterns, SGTF prevalence was lower in individuals living in a care or nursing home (30.7%, compared to 48.2% among those in standard residential accommodation). SGTF prevalence by self-identified ethnicity was 46.6% in the White group, 47.4% in the Asian group, 62.1% in the Black group, and 55.8% in the other, mixed, or unknown ethnicity group. SGTF prevalence was lowest in the most deprived IMD decile (34.1%) and highest in the least deprived decile (53.7%). The prevalence of SGTF among those tested also increased steeply over time (Fig. 1a), ranging from 4.9% during 1–14 November 2020 to 78.7% during 27 December 2020–11 January 2021. Table 2 presents deaths within 28 days of a positive test among study subjects, Table 3 presents crude death rates within 28 days of a positive test per 10,000 person-days of follow-up, and Table 4 for unlimited follow-up (i.e. not restricted to 28 days); the maximum observed follow-up was 71 days. 2,967 individuals in the sample are known to have died, 2,091 of whom died within 28 days of their first positive test (Fig. 1b).

60-day survival assessed by Kaplan-Meier curves was higher in the SGTF group over the entire study sample (Fig. 1c), but this apparently better survival for SGTF individuals is reversed when stratifying the sample by age (Fig. 1d,e) indicating that the association between SGTF and survival is confounded by the younger average age of SGTF cases. Stratifying by broad age groups and by sex, place of residence, ethnicity, index of multiple deprivation, NHS England region, and specimen date, crude death rates within 28 days of a positive SARS-CoV-2 test are higher among SGTF than non-SGTF in 79 of the 104 stratifications assessed (76%; Figs. 1f–k). Stratified death rates are considerably higher in older age groups and in individuals who live in a care or nursing home, and generally, are higher in males than in females, higher in individuals of Asian or Black ethnicity than in individuals of White or Mixed/unknown/other ethnicity, and higher in the most deprived IMD deciles.

## Cox regression analyses

**Fig. 2.**
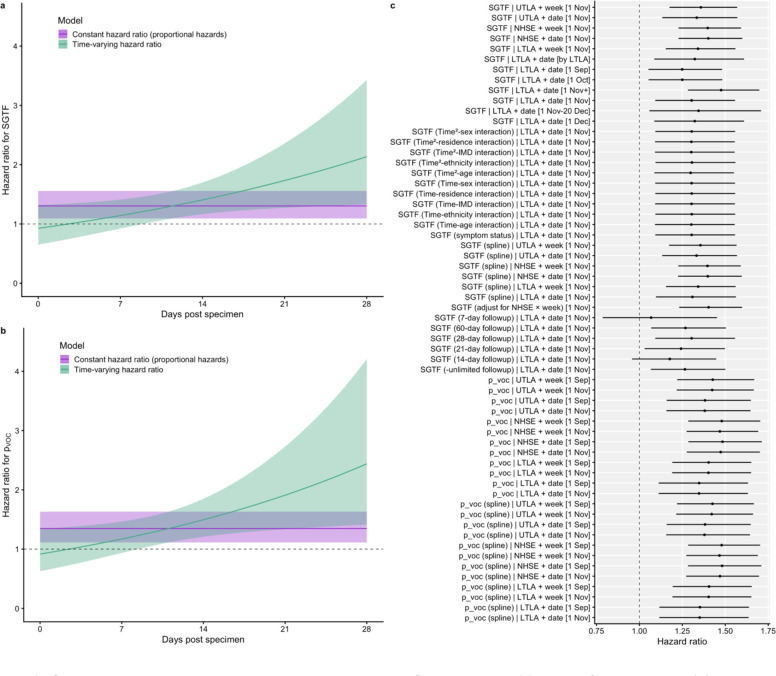
Survival analyses. **a, b** Estimated hazard ratio of death within 28 days of positive test (**a**) using SGTF and (**b**) using variant of concern (VOC) 202012/01 as estimated by our misclassification analysis, in model stratified by LTLA and specimen date and adjusted for the other covariates. **c** Estimated hazard ratio of death within 28 days of positive test across each model tested. Model structures are coded as follows: VOC marker (special features) | stratification [date range]. VOC marker is either SGTF or p_voc. Special features: adjust for NHSE × week, NHS England region and specimen week included as fixed-effect covariates instead of in stratification of the baseline hazard; spline, age and IMD included as spline instead of as linear terms; symptom status, asymptomatic indicator included as a covariate; *X*-day followup, a followup length of *X* days is used instead of the default 28 days; Time-Y interaction, interaction terms included between followup time (optionally: also time^2^) and covariate *Y*. Stratification of the baseline hazard in different models includes geographical level (NHSE: NHS England region; UTLA: upper-tier local authority; LTLA: lower-tier local authority). Date range: Specimens included from the indicated date (in 2020) forward; “by LTLA” signifies a start date chosen separately for each LTLA (see Methods), “1 Nov+” indicates no registration cutoff was used, and “1 Nov-20 Dec” uses an early cutoff so that all individuals have at least 28 days of followup time. Point estimates and 95% confidence intervals shown.

To estimate the effect of SGTF on mortality while controlling for observed confounding, we fitted a series of Cox proportional hazards models^1^ to the data. In the Cox model with baseline hazard stratified by lower tier local authority (LTLA) and specimen date and adjusted for the other covariates, the estimated hazard ratio for SGTF was 1.30 (95% CI 1.09–1.56), indicating that the hazard of death within 28 days of a positive test is 30% (9–56%) higher in those with SGTF compared to non-SGTF (Fig. 2a). Without restricting the length of followup time, we estimated a hazard ratio of 1.26 (1.07–1.50). Including age and IMD as restricted cubic splines rather than linear terms did not change the results. An interaction between SGTF and time was included in the model as an assessment of the proportional hazards assumption. There was strong evidence of non-proportionality of hazards (likelihood ratio test *P* = 0.029; Fig. 2a; Fig. S11). The hazard ratio is just below 1 initially, with confidence intervals including 1, and then increases over time, crossing 1 at 3 days post positive test. The estimated time-varying hazard ratio is 0.95 (0.69–1.33) 1 day after the positive test, 1.14 (0.92–1.42) on day 7, 1.41 (1.16–1.70) on day 14, 1.73 (1.27–2.36) on day 21 and 2.13 (1.33–3.42) on day 28. There was no evidence that adding higher order functions of time into the interaction terms improved model fit (likelihood ratio test *P* = 0.371), and no evidence of a significant interaction between time and age (*P* = 0.609), time and sex (*P* = 0.163), time and IMD (*P* = 0.703), time and ethnicity (*P* = 0.406), or time and residence type (*P* = 0.609).

We found no evidence of a significant interaction between SGTF and age group (likelihood ratio test *P* = 0.804), sex (*P* = 0.543), IMD (*P* = 0.512), and ethnicity (*P* = 0.210). There was weak evidence of an interaction between SGTF and residence type (*P* = 0.0574), with the associated hazard ratio for SGTF being 1.26 (1.05–1.51) in residential settings (houses, flats, sheltered accommodation and houses in multiple occupancy), 2.54 (1.38–4.68) in care/nursing homes, and 1.64 (0.61–3.39) in “other” residence types (i.e. residential institutions including residential education, prisons and detention centres, medical facilities, no fixed abode and other/unknown).

## Misclassification analysis

Prior to the emergence of VOC 202012/01, a number of minor circulating SARS-CoV-2 lineages with spike mutations could also cause SGTF. We restrict our main analyses to specimens from 1 November 2020 onwards to minimise the number of these non-VOC 202012/01 lineages among SGTF-positive samples. However, the appearance of non-VOC 202012/01 samples in SGTF may dilute the estimated effect of VOC 202012/01 on the hazard of mortality, while the exclusion of any VOC 202012/01 samples taken from prior to 1 November 2020 may reduce the power of the analysis. We therefore undertook a misclassification analysis, modelling the relative frequency of SGTF over time for each NHS England region as a combination of a low, time-invariant frequency of non-VOC 202012/01 samples with SGTF plus a logistically growing frequency of VOC 202012/01 samples with SGTF, which allows us to assign to each SGTF sample a probability *p*_VOC_ that the sample is VOC 202012/01 based upon its specimen date and NHS England region (Fig. S12). Again restricting the analysis to specimens from 1 November 2020 onward, we find a hazard ratio associated with *p*_VOC_ of 1.35 (1.11–1.63), slightly higher than the hazard ratio associated with SGTF of 1.30 (1.09–1.56). Including all specimens from 1 September 2020 onward, the hazard ratio associated with *p*_VOC_ remains at 1.35 (1.11–1.63), while the hazard associated with SGTF for the same time period decreases to 1.25 (1.05–1.48).

## Absolute risks

To estimate absolute risks for individuals with VOC 202012/01, we applied the 28- and 60-day hazard ratios obtained for SGTF and *p*_VOC_ to the baseline (i.e. not variant-specific) risk estimated from all specimens taken between 1 August and 31 October 2020 (Table 5). The absolute risk remains low amongst age groups less than 54, and the absolute risk was higher in males compared to females. For SGTF, in females aged 70–84 the risk of death within 28 days increased from 2.9% to 3.7% (95% CI 3.1–4.4%) and for females 85 or older increased from 12.8% to 16.4% (13.7–19.0%). For males aged 70–84 the risk of death within 28 days increased from 4.7% to 6.1% (5.0–7.1%) and for males 85 or older increased from 17.1% to 21.7% (18.3–25.1%). Estimates of the absolute risk at 60 days were higher than those for 28 days and show similar patterns, with the largest increase of 17.1% to 22.3% (18.6–26.1%) in males 85 years and older. Estimates based on *p*_VOC_ were marginally higher. These estimates reflect a substantial increase in absolute risk amongst older age groups. Note that these estimates do not reflect the infection fatality ratio, but the fatality ratio among people tested in the community, and are thus likely to be higher than the infection fatality rate as many infected individuals will not have been tested.

## Further investigations

We conducted a number of sensitivity analyses to verify the robustness of our results. Our main analyses are stratified by LTLA (*n* = 316) and specimen date. Stratifying instead by upper-tier local authority (UTLA; *n* = 150) or by NHS England region (*n* = 7), or by week rather than by date of specimen, produced similar results, with the effect size of SGTF being greater for coarser strata; stratifying at the coarsest level—by NHS England region and specimen week—yielded a hazard ratio for SGTF of 1.40 (1.23–1.59). Adjusting for, rather than stratifying by, specimen week and NHSE region yielded a similar hazard ratio of 1.40 (1.23–1.60). We interpret the decreased effect size of SGTF for finer stratifications as resulting from controlling better for differences in heterogeneities in the availability of hospital services owing to increased hospital demand as local SGTF prevalence increases. When restricting data to specimens collected from September onwards, October onwards, November onwards, or December onwards, the estimated hazard ratio of SGTF increased (Fig. 2c) as the data period became more restricted, which we interpret as resulting from including fewer non-VOC 202012/01 specimens in the SGTF sample. Removing the administrative cutoff to followup time of 10 days prior to data extraction (i.e. 11 January 2020) increased the hazard ratio of SGTF to 1.48 (1.28–1.70), and restricting the analysis to individuals with at least 28 days’ follow-up yielded a hazard ratio of 1.34 (1.06–1.71). Pillar 2 testing data include an indicator for whether the subject was asymptomatic at the time of requesting the test (or symptomatic, or unknown). Although symptomatic status may lie on the causal pathway between SGTF status and death, we adjusted for symptomatic status as a sensitivity analysis and found that it had no effect on the relative hazard of SGTF (1.30 [1.09–1.56]).

## Discussion

Our analysis focuses on deaths within the first 28 days following a positive test, which could overestimate the change in mortality associated with SGTF if individuals infected by VOC 202012/01 die sooner than individuals infected with preexisting SARS-CoV-2 variants. However, the consistency of results when analysing data with 60 days of followup or unlimited followup (Fig. 2c) suggests that this is not the case. By stratifying on test time and region, we attempted to control for the effects of pressure on health services, which cannot be adjusted for directly, as these lie on the causal pathway between infection and mortality.

We do not identify the mechanism for an increased mortality rate in this analysis. There is some evidence that infections with VOC 202012/01 may be associated with higher viral loads, as measured by Ct values detected during PCR testing of specimens (Fig. S13), although Ct values can be biased during the growth phase of an epidemic^2^. Higher viral loads resulting from infection with VOC 202012/01 may be partly responsible for the observed increase in mortality, partly because they may reduce the efficacy of standard antiviral treatments for COVID-19. The impact of viral load on observed SGTF mortality could be assessed using a mediation analysis, which is outside the remit of this study.

We previously identified that the novel SARS-CoV-2 lineage VOC 202012/01 appears to have a substantially greater transmission rate than preexisting variants of SARS-CoV-2^3^, but could not robustly estimate any increase or decrease in associated disease severity from ecological analysis. The individual-level linked community testing data analysed here suggest that the fatality rate among individuals infected with VOC 202012/01 is higher than that associated with infection by preexisting variants. Crucially, due to the nature of the data currently available, we were only able to assess mortality among individuals who received a positive test for SARS-CoV-2 in the community that was processed at one of the three national Lighthouse laboratories capable of returning an SGTF positive or negative result. Indicators for VOC 202012/01 are not currently available for the vast majority of individuals who die due to COVID-19, as they are first tested in hospital. Accordingly, the evidence we provide here must be contextualised with further study of a larger population sample. Our analysis is consistent with analyses by other groups using different methods to verify the increased risk of death among community-tested individuals with SGTF^4^. Estimates of increased mortality based upon Pillar 2 data will become more robust as test results and mortality outcomes continue to accrue over time, although our analysis using stratified Cox regression, which estimates hazard ratios for mortality by comparing outcomes between individuals with and without SGTF who were tested in the same place and at the same time, would no longer accrue additional information at the point when SGTF becomes effectively fixed in England—which may occur as soon as February 2021 if current trends continue^3^.

## Methods

### Data sources —

We linked three datasets provided by Public Health England: a linelist of all positive tests in England’s “Pillar 2” (community) testing for SARS-CoV-2, containing specimen date and demographic information on the test subject; a linelist of cycle threshold (Ct) values for the ORF1ab, N (nucleocapsid), and S (spike) genes for positive tests that were processed in one of the three national laboratories (Alderley Park, Glasgow, or Milton Keynes) utilising the Thermo Fisher TaqPath COVID-19 assay; and a linelist of all deaths due to COVID-19 in England. We link these datasets using a numeric identifier common to all three datasets. We define S gene target failure (SGTF) as any test with Ct < 30 for ORF1ab and N targets but no detectable S gene; non-SGTF as any test with Ct < 30 for ORF1ab, N, and S targets; and all other tests as inconclusive and excluded from the analysis. Because cycle threshold values are not available for individuals who were not tested in the community, this linked dataset does not allow analysis of individuals who first tested positive in hospital, that is, those who presented to hospital after symptom onset without first being tested in the community. This is the reason why our linked dataset has SGTF status for 47% of all community tests (Table S1) from 1 November 2020–22 January 2021, but only 7.4% of all COVID-19 deaths in England following a positive test in either the community or hospital over the same time period (Table S2).

For our main analysis, we included only tests from after 1 November 2020 to avoid including an excess of tests with SGTF not resulting from infection by VOC 202012/01, and censored individuals who did not die within 28 days of their positive test at 28 days. For individuals with less than 28 days of followup after their positive test, we applied censoring at the date of data extraction minus 10 days to reduce any impact of late reporting of deaths. There were missing data for sex (*n* = 7, <0.01%), age (*n* = 56, <0.01%), and IMD and regional covariates (*n* = 1038, 0.1%). There were no missing specimen dates. Individuals with missing age, sex, or geographical location were excluded. We also excluded individuals from the dataset whose age was recorded as zero, as there were 15,400 age-0 individuals compared to 7,212 age-1 individuals in the dataset, suggesting that many of these age-0 individuals may have been miscoded. Categories for missing ethnicity and missing residence type were created.

We grouped residence types into three categories: Residential, which included the “Residential dwelling (including houses, flats, sheltered accommodation)” and “House in multiple occupancy (HMO)” groups; Care/Nursing home; and Other/Unknown, which included the “Medical facilities (including hospitals and hospices, and mental health)”, “No fixed abode”, “Other property classifications”, “Overseas address”, “Prisons, detention centres, secure units”, “Residential institution (including residential education)”, and “Undetermined” groups, as well as unspecified residence type. We grouped ethnicities into four categories according to the broad categories used in the 2011 UK Census: Asian, which included the “Bangladeshi (Asian or Asian British)”, “Chinese (other ethnic group)”, “Indian (Asian or Asian British)”, “Pakistani (Asian or Asian British)”, and “Any other Asian background” groups; Black, which included the “African (Black or Black British)”, “Caribbean (Black or Black British)”, and “Any other Black background” groups; White, which included the “British (White)”, “Irish (White)”, and “Any other White background” groups; and Other / Mixed / Unknown, which included the “Any other ethnic group”, “White and Asian (Mixed)”, “White and Black African (Mixed)”, “White and Black Caribbean (Mixed)”, “Any other Mixed background”, and “Unknown” groups.

### Statistical methods —

There are several factors that we expect to be associated with both the probability of SGTF and with risk of death, thus confounding the association between SGTF and risk of death in those tested. Area of residence and specimen date were expected to be potentially strong confounders. Area of residence is expected to be strongly associated with SGTF status due to different virus variants circulating in different areas, and specimen date because the prevalence of SGTF is known to have greatly increased over time. Area of residence and specimen date are also expected to be associated with risk of death following a test, including due to differential pressure on hospital resources by area and time. The following variables were also identified as potential confounders: sex, age, place of residence (Residential, Care/Nursing home, or Other/Unknown), ethnicity (White, Asian, Black, or Other/Mixed/Unknown), index of multiple deprivation (IMD, in deciles).

Descriptive analyses were performed. We tabulated the association between SGTF and each of the potential confounders (Table 1). Missing data in the exposure by levels of the confounders were tabulated (Table S1). The unadjusted association between SGTF and mortality was also assessed using a Kaplan-Meier plot and by tabulating mortality rates. Kaplan-Meier plots (Figs. S1–S10) and mortality rates (Tables 4–5) are also presented separately according to categories of the potential confounders. Exact Poisson CIs are used for mortality rates, assuming constant rate.

We performed complete cases analysis. This assumes that for each analysis, the missing data are independent from the outcome of interest, given the variables included in the models. We also assumed that censoring is uninformative, which is plausible as all censoring is administrative.

Cox regression was used to estimate the association between SGTF and the hazard for mortality, conditioning on the potential confounders listed above. The baseline hazard in the Cox model was stratified by both specimen date and LTLA, therefore finely controlling for these variables. The remaining variables were included as covariates in the model (sex, age, place of residence, ethnicity, IMD decile). Age and IMD were included either as linear terms, given an observed log-linear effect of age on the infection fatality rate of SARS-CoV-2^5^, or as restricted cubic splines with 3 knots. The time origin for the analysis was specimen date and we considered deaths up to 28 days after the specimen date. Individuals who did not die within 28 days were censored at the earlier of 28 days post specimen date and the administrative censoring date, which we chose as the date of the most recent death linkable to SGTF status minus 10 days in order to minimise any potential bias due to late reporting of deaths. We began by assuming proportionality of hazards for SGTF and the covariates included in the model. The proportional hazards assumption was assessed by including in the model an interaction between each covariate and time, which was performed separately for SGTF and for each other covariate. Schoenfeld residual plots were also obtained for each covariate. We assessed whether the association between SGTF and the hazard was modified by age, sex, IMD, ethnicity, and place of residence. Models with and without interactions were compared using likelihood ratio tests.

### Misclassification analysis —

The exposure of SGTF is subject to misclassification, because a number of minor circulating variants of SARS-CoV-2 in addition to VOC 202012/01 are also associated with failure to amplify the spike gene target. Accordingly, a positive test with SGTF is not necessarily indicative of infection with VOC 202012/01. Misclassification of an exposure can result in bias in its estimated association with the outcome. We fitted a beta-binomial logistic growth model to Pillar 2 data by NHS region to estimate a “background” rate of SGTF in the absence of VOC 202012/01. This model is then used to estimate the probability that an individual testing positive with SGTF is infected with VOC 202012/01, separately for individuals in each NHS region. These probabilities can then be used in place of the binary SGTF exposure in the Cox models (assuming proportional hazards, and allowing for a time-varying hazard ratio where there is evidence of proportional hazards violations).

We fitted models of logistic growth accounting for false positives (modelled as regionally-varying background rates of SGTF associated with non-VOC 202012/01 variants) to the SGTF data. Our logistic beta-binomial model of VOC 202012/01 growth is as follows:

slope ~ normal (μ=0,σ=1)

intercept ~ normal (μ=0,σ=1000)

falsepos ~ beta (α=1.5,β=15)

conc~normal(μ=0,σ=500)≥2

f(t)=exp( slope ×(t− intercept ))/(1+exp( slope ×(t− intercept )))

s(t)=f(t)+(1−f(t))× falsepos 

$$k_{t}\sim \text{ betaBinomial }\left(n=n_{t},\alpha =s(t)\times (\text{conc}-2)+1,\beta =(1-s(t))\times (\text{conc}-2)+1\right)$$

Here, *f*(*t*) is the model-predicted frequency of VOC 202012/01 at time *t* based on the terms *slope* and *intercept*, *s*(*t*) is the model-predicted frequency of S gene target failure at time *t* owing to a background false positive rate *falsepos*, *conc* is the “concentration” parameter (= α + β) of a beta distribution with mode *s*(*t*), *k*_*t*_ is the number of S gene target failures detected at time *t* and *n*_*t*_ is the total number of tests at time *t*.

### Absolute risks —

Estimates from the final Cox models were used to obtain estimates of absolute risk of death for 28 and 60 days with SGTF and *p*_VOC_. Given the strong influence of age on risk of death, we present absolute risks by sex and age group (1–34, 35–54, 55–69, 70–84, 85+). Absolute risks of death (case fatality rate) within 28 and 60 days were estimated by age group and sex using data on individuals tested in August, September, and October 2020; this is referred to as the baseline risk. The absolute risks of death for individuals with SGTF were then estimated as follows. If the baseline absolute risk of death in a given age group is (1 − *A*), then the estimated absolute risk of death with SGTF is (1 − *A*^*HR*^), where HR denotes the estimated hazard ratio obtained from the Cox model with proportional hazards. We applied the hazard ratio from 28 days to the baseline risk for 28 days, and the hazard ratio for 60 days to the baseline risk for 60 days, to estimate absolute risks of death for individuals with SGTF and uncertainty of these estimates. Standard errors are obtained via the delta method, and CIs based on normal approximations.

### Sensitivity analyses —

Several sensitivity analyses were performed. After establishing the final model through using the process outlined above we investigated the impact of using different variables for stratification of the baseline hazard measuring region at a coarser level (UTLA, or NHS England region), as well as coarser test specimen time (week rather than exact date). Adjusting for these variables instead of using stratification was also explored. We also repeated the main analysis restricting data to specimens collected from September onwards, October onwards, November onwards, or December onwards.

To assess the impact of imposing an administrative cutoff to follow-up time of 10 days prior to data extraction, we first reanalysed the data without this cutoff, as well as reanalysing the data restricting the analysis to individuals with at least 28 days’ follow-up.

Finally, we adjusted for symptomatic status at the time of requesting the test (asymptomatic, symptomatic, or unknown).

## Supplementary Material

1

## Figures and Tables

**Table 1.** Characteristics of study subjects and prevalence of SGTF among study subjects.

	All	SGTF	Non-SGTF	SGTF prevalence
	858,181 (100%)	411,961 (100%)	446,220 (100%)	411,961 / 858,181 (48%)
**Sex**				
**Female**	449,688 (52.4%)	213,374 (51.8%)	236,314 (53%)	213,374 / 449,688 (47.4%)
**Male**	408,493 (47.6%)	198,587 (48.2%)	209,906 (47%)	198,587 / 408,493 (48.6%)
**Age**				
**1–34**	389,154 (45.3%)	188,967 (45.9%)	200,187 (44.9%)	188,967 / 389,154 (48.6%)
**35–54**	301,960 (35.2%)	147,747 (35.9%)	154,213 (34.6%)	147,747 / 301,960 (48.9%)
**55–69**	128,844 (15%)	59,664 (14.5%)	69,180 (15.5%)	59,664 / 128,844 (46.3%)
**70–84**	32,472 (3.8%)	13,495 (3.3%)	18,977 (4.3%)	13,495 / 32,472 (41.6%)
**85 and older**	5,751 (0.7%)	2,088 (0.5%)	3,663 (0.8%)	2,088 / 5,751 (36.3%)
**Place of residence**				
**Residential**	824,880 (96.1%)	397,203 (96.4%)	427,677 (95.8%)	397,203 / 824,880 (48.2%)
**Care/Nursing home**	4,669 (0.5%)	1,435 (0.3%)	3,234 (0.7%)	1,435 / 4,669 (30.7%)
**Other/Unknown**	28,632 (3.3%)	13,323 (3.2%)	15,309 (3.4%)	13,323 / 28,632 (46.5%)
**Ethnicity**				
**White**	638,520 (74.4%)	297,863 (72.3%)	340,657 (76.3%)	297,863 / 638,520 (46.6%)
**Asian**	125,676 (14.6%)	59,512 (14.4%)	66,164 (14.8%)	59,512 / 125,676 (47.4%)
**Black**	33,848 (3.9%)	21,028 (5.1%)	12,820 (2.9%)	21,028 / 33,848 (62.1%)
**Other/Mixed/Unknown**	60,137 (7%)	33,558 (8.1%)	26,579 (6%)	33,558 / 60,137 (55.8%)
**Index of Multiple Deprivation decile**				
**1**	105,211 (12.3%)	35,913 (8.7%)	69,298 (15.5%)	35,913 / 105,211 (34.1%)
**2**	103,249 (12%)	46,668 (11.3%)	56,581 (12.7%)	46,668 / 103,249 (45.2%)
**3**	100,018 (11.7%)	48,777 (11.8%)	51,241 (11.5%)	48,777 / 100,018 (48.8%)
**4**	90,559 (10.6%)	45,849 (11.1%)	44,710 (10%)	45,849 / 90,559 (50.6%)
**5**	84,802 (9.9%)	43,639 (10.6%)	41,163 (9.2%)	43,639 / 84,802 (51.5%)
**6**	78,761 (9.2%)	40,317 (9.8%)	38,444 (8.6%)	40,317 / 78,761 (51.2%)
**7**	77,367 (9%)	38,565 (9.4%)	38,802 (8.7%)	38,565 / 77,367 (49.8%)
**8**	77,017 (9%)	38,050 (9.2%)	38,967 (8.7%)	38,050 / 77,017 (49.4%)
**9**	74,305 (8.7%)	38,257 (9.3%)	36,048 (8.1%)	38,257 / 74,305 (51.5%)
**10**	66,892 (7.8%)	35,926 (8.7%)	30,966 (6.9%)	35,926 / 66,892 (53.7%)
**NHS England region**				
**East of England**	75,614 (8.8%)	54,339 (13.2%)	21,275 (4.8%)	54,339 / 75,614 (71.9%)
**London**	166,115 (19.4%)	117,899 (28.6%)	48,216 (10.8%)	117,899 / 166,115 (71%)
**Midlands**	169,911 (19.8%)	61,254 (14.9%)	108,657 (24.4%)	61,254 / 169,911 (36.1%)
**North East and Yorkshire**	157,965 (18.4%)	37,400 (9.1%)	120,565 (27%)	37,400 / 157,965 (23.7%)
**North West**	139,909 (16.3%)	43,112 (10.5%)	96,797 (21.7%)	43,112 / 139,909 (30.8%)
**South East**	120,712 (14.1%)	86,656 (21%)	34,056 (7.6%)	86,656 / 120,712 (71.8%)
**South West**	27,955 (3.3%)	11,301 (2.7%)	16,654 (3.7%)	11,301 / 27,955 (40.4%)
**Specimen date**				
**1 Nov-14 Nov**	164,495 (19.2%)	8,029 (1.9%)	156,466 (35.1%)	8,029 / 164,495 (4.9%)
**15 Nov-28 Nov**	111,198 (13%)	12,243 (3%)	98,955 (22.2%)	12,243 / 111,198 (11%)
**29 Nov-12 Dec**	104,865 (12.2%)	37,910 (9.2%)	66,955 (15%)	37,910 / 104,865 (36.2%)
**13 Dec-26 Dec**	169,491 (19.8%)	111,367 (27%)	58,124 (13%)	111,367 / 169,491 (65.7%)
**27 Dec-11 Jan**	308,132 (35.9%)	242,412 (58.8%)	65,720 (14.7%)	242,412 / 308,132 (78.7%)

**Table 2.** Deaths within 28 days of a positive test among study subjects.

	All	SGTF	Non-SGTF
	2,091 (100%)	687 (100%)	1,404 (100%)
**Sex**			
**Female**	895 (42.8%)	283 (41.2%)	612 (43.6%)
**Male**	1,196 (57.2%)	404 (58.8%)	792 (56.4%)
**Age**			
**1–34**	19 (0.9%)	6 (0.9%)	13 (0.9%)
**35–54**	141 (6.7%)	57 (8.3%)	84 (6%)
**55–69**	435 (20.8%)	173 (25.2%)	262 (18.7%)
**70–84**	869 (41.6%)	287 (41.8%)	582 (41.5%)
**85 and older**	627 (30%)	164 (23.9%)	463 (33%)
**Place of residence**			
**Residential**	1,569 (75%)	547 (79.6%)	1,022 (72.8%)
**Care/Nursing home**	475 (22.7%)	123 (17.9%)	352 (25.1%)
**Other/Unknown**	47 (2.2%)	17 (2.5%)	30 (2.1%)
**Ethnicity**			
**White**	1,705 (81.5%)	532 (77.4%)	1,173 (83.5%)
**Asian**	278 (13.3%)	109 (15.9%)	169 (12%)
**Black**	54 (2.6%)	28 (4.1%)	26 (1.9%)
**Other/Mixed/Unknown**	54 (2.6%)	18 (2.6%)	36 (2.6%)
**Index of Multiple Deprivation decile**			
**1**	341 (16.3%)	72 (10.5%)	269 (19.2%)
**2**	281 (13.4%)	88 (12.8%)	193 (13.7%)
**3**	225 (10.8%)	53 (7.7%)	172 (12.3%)
**4**	206 (9.9%)	81 (11.8%)	125 (8.9%)
**5**	176 (8.4%)	70 (10.2%)	106 (7.5%)
**6**	196 (9.4%)	81 (11.8%)	115 (8.2%)
**7**	157 (7.5%)	61 (8.9%)	96 (6.8%)
**8**	184 (8.8%)	73 (10.6%)	111 (7.9%)
**9**	171 (8.2%)	56 (8.2%)	115 (8.2%)
**10**	154 (7.4%)	52 (7.6%)	102 (7.3%)
**NHS England region**			
**East of England**	151 (7.2%)	85 (12.4%)	66 (4.7%)
**London**	229 (11%)	162 (23.6%)	67 (4.8%)
**Midlands**	445 (21.3%)	80 (11.6%)	365 (26%)
**North East and Yorkshire**	603 (28.8%)	91 (13.2%)	512 (36.5%)
**North West**	333 (15.9%)	64 (9.3%)	269 (19.2%)
**South East**	248 (11.9%)	187 (27.2%)	61 (4.3%)
**South West**	82 (3.9%)	18 (2.6%)	64 (4.6%)
**Specimen date**			
**1 Nov-14 Nov**	548 (26.2%)	20 (2.9%)	528 (37.6%)
**15 Nov-28 Nov**	339 (16.2%)	26 (3.8%)	313 (22.3%)
**29 Nov-12 Dec**	390 (18.7%)	124 (18%)	266 (18.9%)
**13 Dec-26 Dec**	475 (22.7%)	281 (40.9%)	194 (13.8%)
**27 Dec-11 Jan**	339 (16.2%)	236 (34.4%)	103 (7.3%)

**Table 3.** Rates of death within 28 days of positive test among study subjects. Total number of deaths, number of days of followup, and deaths per 10,000 days of followup reported.

	All	SGTF	Non-SGTF
	2,091 / 16,776,166 (1.25)	687 / 5,882,304 (1.17)	1,404 / 10,893,861 (1.29)
**Sex**			
**Female**	895 / 8,823,354 (1.01)	283 / 3,055,682 (0.93)	612 / 5,767,671 (1.06)
**Male**	1,196 / 7,952,812 (1.5)	404 / 2,826,622 (1.43)	792 / 5,126,190 (1.55)
**Age**			
**1–34**	19 / 7,693,867 (0.02)	6 / 2,767,823 (0.02)	13 / 4,926,044 (0.03)
**35–54**	141 / 5,907,410 (0.24)	57 / 2,133,536 (0.27)	84 / 3,773,873 (0.22)
**55–69**	435 / 2,458,532 (1.77)	173 / 789,704 (2.19)	262 / 1,668,828 (1.57)
**70–84**	869 / 615,306 (14.12)	287 / 168,523 (17.03)	582 / 446,784 (13.03)
**85 and older**	627 / 101,050 (62.05)	164 / 22,718 (72.19)	463 / 78,332 (59.11)
**Place of residence**			
**Residential**	1,569 / 16,108,070 (0.97)	547 / 5,668,687 (0.96)	1,022 / 10,439,383 (0.98)
**Care/Nursing home**	475 / 87,001 (54.6)	123 / 17,672 (69.6)	352 / 69,329 (50.77)
**Other/Unknown**	47 / 581,094 (0.81)	17 / 195,946 (0.87)	30 / 385,149 (0.78)
**Ethnicity**			
**White**	1,705 / 12,541,963 (1.36)	532 / 4,275,094 (1.24)	1,173 / 8,266,870 (1.42)
**Asian**	278 / 2,484,370 (1.12)	109 / 827,494 (1.32)	169 / 1,656,876 (1.02)
**Black**	54 / 598,024 (0.9)	28 / 286,850 (0.98)	26 / 311,174 (0.84)
**Other/Mixed/Unknown**	54 / 1,151,809 (0.47)	18 / 492,868 (0.37)	36 / 658,941 (0.55)
**Index of Multiple Deprivation decile**			
**1**	341 / 2,061,034 (1.65)	72 / 393,858 (1.83)	269 / 1,667,175 (1.61)
**2**	281 / 2,003,138 (1.4)	88 / 622,894 (1.41)	193 / 1,380,244 (1.4)
**3**	225 / 1,943,584 (1.16)	53 / 689,400 (0.77)	172 / 1,254,184 (1.37)
**4**	206 / 1,746,970 (1.18)	81 / 652,995 (1.24)	125 / 1,093,974 (1.14)
**5**	176 / 1,645,732 (1.07)	70 / 642,930 (1.09)	106 / 1,002,802 (1.06)
**6**	196 / 1,539,366 (1.27)	81 / 598,790 (1.35)	115 / 940,576 (1.22)
**7**	157 / 1,528,348 (1.03)	61 / 577,502 (1.06)	96 / 950,846 (1.01)
**8**	184 / 1,517,480 (1.21)	73 / 565,228 (1.29)	111 / 952,252 (1.17)
**9**	171 / 1,473,780 (1.16)	56 / 586,332 (0.96)	115 / 887,449 (1.3)
**10**	154 / 1,316,735 (1.17)	52 / 552,376 (0.94)	102 / 764,360 (1.33)
**NHS England region**			
**East of England**	151 / 1,389,416 (1.09)	85 / 865,050 (0.98)	66 / 524,366 (1.26)
**London**	229 / 3,208,122 (0.71)	162 / 1,976,046 (0.82)	67 / 1,232,076 (0.54)
**Midlands**	445 / 3,314,751 (1.34)	80 / 635,712 (1.26)	365 / 2,679,039 (1.36)
**North East and Yorkshire**	603 / 3,393,368 (1.78)	91 / 418,625 (2.17)	512 / 2,974,742 (1.72)
**North West**	333 / 2,603,784 (1.28)	64 / 372,149 (1.72)	269 / 2,231,635 (1.21)
**South East**	248 / 2,327,066 (1.07)	187 / 1,472,920 (1.27)	61 / 854,146 (0.71)
**South West**	82 / 539,660 (1.52)	18 / 141,804 (1.27)	64 / 397,856 (1.61)
**Specimen date**			
**1 Nov-14 Nov**	548 / 4,598,236 (1.19)	20 / 224,551 (0.89)	528 / 4,373,686 (1.21)
**15 Nov-28 Nov**	339 / 3,108,314 (1.09)	26 / 342,473 (0.76)	313 / 2,765,842 (1.13)
**29 Nov-12 Dec**	390 / 2,930,586 (1.33)	124 / 1,059,924 (1.17)	266 / 1,870,662 (1.42)
**13 Dec-26 Dec**	475 / 3,806,607 (1.25)	281 / 2,463,718 (1.14)	194 / 1,342,890 (1.44)
**27 Dec-11 Jan**	339 / 2,332,422 (1.45)	236 / 1,791,640 (1.32)	103 / 540,782 (1.9)

**Table 4.** Rates of death within any time period following positive test among study subjects. Total number of deaths, number of days of followup, and deaths per 10,000 days of followup reported.

	All	SGTF	Non-SGTF
	2,315 / 26,176,060 (0.88)	705 / 6,696,880 (1.05)	1,610 / 19,479,180 (0.83)
**Sex**			
**Female**	988 / 13,786,478 (0.72)	290 / 3,481,554 (0.83)	698 / 10,304,925 (0.68)
**Male**	1,327 / 12,389,581 (1.07)	415 / 3,215,326 (1.29)	912 / 9,174,255 (0.99)
**Age**			
**1–34**	23 / 11,944,753 (0.02)	6 / 3,162,130 (0.02)	17 / 8,782,623 (0.02)
**35–54**	164 / 9,153,070 (0.18)	60 / 2,421,846 (0.25)	104 / 6,731,224 (0.15)
**55–69**	510 / 3,918,577 (1.3)	178 / 895,650 (1.99)	332 / 3,022,926 (1.1)
**70–84**	942 / 1,000,954 (9.41)	293 / 191,878 (15.27)	649 / 809,076 (8.02)
**85 and older**	676 / 158,706 (42.59)	168 / 25,376 (66.21)	508 / 133,331 (38.1)
**Place of residence**			
**Residential**	1,758 / 25,106,189 (0.7)	560 / 6,451,174 (0.87)	1,198 / 18,655,015 (0.64)
**Care/Nursing home**	508 / 141,083 (36.01)	127 / 20,434 (62.15)	381 / 120,649 (31.58)
**Other/Unknown**	49 / 928,788 (0.53)	18 / 225,272 (0.8)	31 / 703,516 (0.44)
**Ethnicity**			
**White**	1,893 / 19,790,819 (0.96)	548 / 4,894,532 (1.12)	1,345 / 14,896,288 (0.9)
**Asian**	303 / 3,832,494 (0.79)	109 / 926,274 (1.18)	194 / 2,906,220 (0.67)
**Black**	61 / 851,538 (0.72)	30 / 322,224 (0.93)	31 / 529,314 (0.59)
**Other/Mixed/Unknown**	58 / 1,701,208 (0.34)	18 / 553,850 (0.32)	40 / 1,147,358 (0.35)
**Index of Multiple Deprivation decile**			
**1**	380 / 3,465,168 (1.1)	73 / 454,148 (1.61)	307 / 3,011,020 (1.02)
**2**	312 / 3,185,588 (0.98)	90 / 707,200 (1.27)	222 / 2,478,388 (0.9)
**3**	244 / 3,017,818 (0.81)	53 / 784,698 (0.68)	191 / 2,233,120 (0.86)
**4**	225 / 2,688,858 (0.84)	84 / 741,152 (1.13)	141 / 1,947,706 (0.72)
**5**	200 / 2,519,846 (0.79)	72 / 735,742 (0.98)	128 / 1,784,104 (0.72)
**6**	214 / 2,352,866 (0.91)	82 / 682,912 (1.2)	132 / 1,669,954 (0.79)
**7**	181 / 2,362,076 (0.77)	62 / 659,002 (0.94)	119 / 1,703,074 (0.7)
**8**	199 / 2,348,292 (0.85)	76 / 642,545 (1.18)	123 / 1,705,746 (0.72)
**9**	190 / 2,245,194 (0.85)	58 / 663,816 (0.87)	132 / 1,581,377 (0.83)
**10**	170 / 1,990,355 (0.85)	55 / 625,664 (0.88)	115 / 1,364,690 (0.84)
**NHS England region**			
**East of England**	168 / 1,842,747 (0.91)	87 / 960,092 (0.91)	81 / 882,656 (0.92)
**London**	244 / 4,322,423 (0.56)	167 / 2,219,614 (0.75)	77 / 2,102,810 (0.37)
**Midlands**	500 / 5,358,479 (0.93)	82 / 702,650 (1.17)	418 / 4,655,829 (0.9)
**North East and Yorkshire**	680 / 6,080,990 (1.12)	94 / 489,701 (1.92)	586 / 5,591,290 (1.05)
**North West**	372 / 4,457,542 (0.83)	64 / 399,443 (1.6)	308 / 4,058,099 (0.76)
**South East**	263 / 3,250,598 (0.81)	193 / 1,760,200 (1.1)	70 / 1,490,397 (0.47)
**South West**	88 / 863,280 (1.02)	18 / 165,180 (1.09)	70 / 698,100 (1)
**Specimen date**			
**1 Nov-14 Nov**	676 / 10,581,858 (0.64)	23 / 509,274 (0.45)	653 / 10,072,584 (0.65)
**15 Nov-28 Nov**	400 / 5,691,072 (0.7)	32 / 609,924 (0.52)	368 / 5,081,148 (0.72)
**29 Nov-12 Dec**	425 / 3,752,496 (1.13)	133 / 1,315,496 (1.01)	292 / 2,437,001 (1.2)
**13 Dec-26 Dec**	475 / 3,818,211 (1.24)	281 / 2,470,546 (1.14)	194 / 1,347,664 (1.44)
**27 Dec-11 Jan**	339 / 2,332,422 (1.45)	236 / 1,791,640 (1.32)	103 / 540,782 (1.9)

**Table 5.** Absolute risk associated with SGTF and *p*_VOC_, as expressed by case fatality ratio (%) among individuals testing positive in the community. The baseline absolute risk after 28 days and 60 days post-test is derived using linked deaths for all individuals testing positive in the community from 1 August–31 October 2020.

Sex	Age	Baseline, 28 days	SGTF, 28 days	pVOC, 28 days	Baseline, 60 days	SGTF, 60 days	pVOC, 60 days
**Female**	0–34	0.00069 (0.00069–0.00069)	0.000900 (0.000741–0.00106)	0.000931 (0.000752–0.00111)	0.00138 (0.00138–0.00138)	0.00175 (0.00145–0.00205)	0.00182 (0.00148–0.00216)
**Female**	35–54	0.0326 (0.0326–0.0326)	0.0425 (0.035–0.0501)	0.0439 (0.0355–0.0524)	0.0396 (0.0396–0.0396)	0.0502 (0.0416–0.0588)	0.0521 (0.0424–0.0618)
**Female**	55–69	0.183 (0.183–0.183)	0.238 (0.196–0.28)	0.246 (0.199–0.293)	0.254 (0.254–0.254)	0.322 (0.267–0.377)	0.334 (0.272–0.396)
**Female**	70–84	2.88 (2.88–2.88)	3.74 (3.09–4.39)	3.86 (3.14–4.59)	3.43 (3.43–3.43)	4.33 (3.61–5.06)	4.49 (3.67–5.31)
**Female**	85 and older	12.8 (12.8–12.8)	16.4 (13.7–19)	16.8 (13.9–19.8)	15.9 (15.8–15.9)	19.7 (16.6–22.7)	20.3 (16.9–23.7)
**Male**	0–34	0.00306 (0.00306–0.00306)	0.00399 (0.00329–0.0047)	0.00413 (0.00333–0.00492)	0.00459 (0.00459–0.00459)	0.00582 (0.00483–0.00682)	0.00605 (0.00492–0.00717)
**Male**	35–54	0.0627 (0.0627–0.0627)	0.0818 (0.0673–0.0963)	0.0845 (0.0683–0.101)	0.0867 (0.0867–0.0868)	0.11 (0.0912–0.129)	0.114 (0.0928–0.135)
**Male**	55–69	0.559 (0.559–0.56)	0.729 (0.6–0.858)	0.753 (0.609–0.897)	0.734 (0.734–0.734)	0.93 (0.772–1.09)	0.965 (0.785–1.14)
**Male**	70–84	4.7 (4.7–4.7)	6.08 (5.04–7.12)	6.28 (5.11–7.44)	5.62 (5.62–5.62)	7.07 (5.91–8.24)	7.33 (6.02–8.65)
**Male**	85 and older	17.1 (17.1–17.1)	21.7 (18.3–25.1)	22.3 (18.6–26.1)	20.5 (20.4–20.5)	25.2 (21.5–28.9)	26 (21.9–30.2)
