# Supplementary material for: Increased mortality in community-tested cases of SARS-CoV-2 lineage B.1.1.7
Source: medRxiv. 2021 Mar 5:2021.02.01.21250959. Originally published 2021 Feb 3. Preprint. [Version 3] doi: 10.1101/2021.02.01.21250959 (PMC7872389; doi:10.1101/2021.02.01.21250959)
Supplement: 1 [file NIHPP2021.02.01.21250959-supplement-1.pdf]

medRxiv preprint doi: https://doi.org/10.1101/2021.02.01.21250959; this version posted March 5, 2021. The copyright holder for this preprint (which was not certified by peer review) is the author/funder, who has granted medRxiv a license to display the preprint in perpetuity.
It is made available under a CC-BY 4.0 International license .

Supplementary Information for Increased mortality in community-tested cases of SARS-CoV-2 lineage B.1.1.7

Nicholas G. Davies, Christopher I. Jarvis, CMMID COVID-19 Working Group,
W. John Edmunds, Nicholas P. Jewell, Karla Diaz-Ordaz, Ruth H. Keogh

Contents

Supplementary Tables 1–2
Supplementary Notes 1–2

medRxiv preprint doi: https://doi.org/10.1101/2021.02.01.21250959; this version posted March 5, 2021. The copyright holder for this preprint (which was not certified by peer review) is the author/funder, who has granted medRxiv a license to display the preprint in perpetuity.
It is made available under a CC-BY 4.0 International license .

Supplementary Table 1. Rates of death within any time period following positive test among study subjects, including missing SGTF status. Total number of deaths, number of days of followup, and deaths per 10,000 days of followup reported. The maximum observed followup was 105 days.

	All	Missing	SGTF	Non-SGTF
	19,615 / 111,457,728 (1.76)	13,989 / 50,338,187 (2.78)	3,362 / 25,927,734 (1.3)	2,264 / 35,191,806 (0.64)
**Sex**				
**Female**	10,393 / 59,964,454 (1.73)	7,923 / 27,867,530 (2.84)	1,461 / 13,457,980 (1.09)	1,009 / 18,638,942 (0.54)
**Male**	9,222 / 51,493,274 (1.79)	6,066 / 22,470,656 (2.7)	1,901 / 12,469,754 (1.52)	1,255 / 16,552,864 (0.76)
**Age**				
**1-34**	84 / 50,157,768 (0.02)	32 / 22,443,774 (0.01)	29 / 11,879,958 (0.02)	23 / 15,834,034 (0.01)
**35-54**	856 / 38,321,232 (0.22)	398 / 16,839,060 (0.24)	309 / 9,307,178 (0.33)	149 / 12,174,994 (0.12)
**55-69**	2,641 / 16,670,620 (1.58)	1,285 / 7,431,238 (1.73)	903 / 3,767,472 (2.4)	453 / 5,471,911 (0.83)
**70-84**	6,528 / 4,629,068 (14.1)	4,413 / 2,335,022 (18.9)	1,215 / 832,234 (14.6)	900 / 1,461,813 (6.16)
**85 and older**	9,506 / 1,679,038 (56.62)	7,861 / 1,289,092 (60.98)	906 / 140,892 (64.3)	739 / 249,054 (29.67)
**Place of residence**				
**Residential**	7,796 / 104,825,376 (0.74)	3,550 / 45,778,629 (0.78)	2,606 / 25,079,712 (1.04)	1,640 / 33,967,036 (0.48)
**Care/Nursing home**	11,472 / 3,134,891 (36.59)	10,183 / 2,792,355 (36.47)	692 / 115,209 (60.06)	597 / 227,327 (26.26)
**Other/Unknown**	347 / 3,497,460 (0.99)	256 / 1,767,203 (1.45)	64 / 732,813 (0.87)	27 / 997,444 (0.27)
**Ethnicity**				
**White**	17,272 / 82,953,286 (2.08)	12,635 / 37,068,638 (3.41)	2,704 / 18,966,734 (1.43)	1,933 / 26,917,914 (0.72)
**Asian**	1,417 / 15,309,440 (0.93)	731 / 6,447,530 (1.13)	439 / 3,648,430 (1.2)	247 / 5,213,480 (0.47)
**Black**	377 / 4,801,714 (0.79)	229 / 2,569,738 (0.89)	113 / 1,251,373 (0.9)	35 / 980,604 (0.36)
**Other/Mixed/Unknown**	549 / 8,393,288 (0.65)	394 / 4,252,282 (0.93)	106 / 2,061,196 (0.51)	49 / 2,079,810 (0.24)
**IMD decile**				
**1-2 (most deprived)**	3,871 / 25,401,898 (1.52)	2,239 / 10,049,132 (2.23)	884 / 5,407,510 (1.63)	748 / 9,945,257 (0.75)
**3-4**	4,095 / 25,376,552 (1.61)	2,831 / 11,867,324 (2.39)	792 / 5,947,716 (1.33)	472 / 7,561,513 (0.62)
**5-6**	4,091 / 22,075,895 (1.85)	3,128 / 10,566,596 (2.96)	599 / 5,256,524 (1.14)	364 / 6,252,776 (0.58)
**7-8**	3,892 / 20,384,284 (1.91)	2,966 / 9,458,600 (3.14)	573 / 4,782,278 (1.2)	353 / 6,143,406 (0.57)
**9-10**	3,666 / 18,219,098 (2.01)	2,825 / 8,396,536 (3.36)	514 / 4,533,706 (1.13)	327 / 5,288,855 (0.62)
**NHS England region**				
**East of England**	2,748 / 12,982,012 (2.12)	2,347 / 8,084,884 (2.9)	300 / 3,274,839 (0.92)	101 / 1,622,290 (0.62)
**London**	2,122 / 24,247,196 (0.88)	1,602 / 13,541,876 (1.18)	423 / 6,941,281 (0.61)	97 / 3,764,038 (0.26)
**Midlands**	4,091 / 21,368,735 (1.91)	2,790 / 9,120,507 (3.06)	723 / 3,789,278 (1.91)	578 / 8,458,950 (0.68)
**North East & Yorkshire**	2,536 / 15,708,002 (1.61)	1,133 / 3,225,434 (3.51)	599 / 2,654,326 (2.26)	804 / 9,828,241 (0.82)
**North West**	2,151 / 13,480,134 (1.6)	1,066 / 2,839,457 (3.75)	594 / 3,087,830 (1.92)	491 / 7,552,846 (0.65)
**South East**	4,125 / 17,202,003 (2.4)	3,474 / 9,072,312 (3.83)	552 / 5,446,734 (1.01)	99 / 2,682,958 (0.37)
**South West**	1,842 / 6,469,646 (2.85)	1,577 / 4,453,717 (3.54)	171 / 733,445 (2.33)	94 / 1,282,484 (0.73)
**Specimen date**				
**1 Nov-21 Nov**	2,830 / 33,119,124 (0.85)	1,862 / 11,270,923 (1.65)	37 / 1,243,146 (0.3)	931 / 20,605,054 (0.45)
**22 Nov-12 Dec**	2,481 / 17,290,316 (1.43)	1,794 / 6,249,994 (2.87)	171 / 3,160,206 (0.54)	516 / 7,880,116 (0.65)
**13 Dec-2 Jan**	4,901 / 33,869,084 (1.45)	3,572 / 18,109,882 (1.97)	915 / 10,943,494 (0.84)	414 / 4,815,710 (0.86)
**3 Jan-23 Jan**	7,959 / 23,635,116 (3.37)	5,864 / 12,863,158 (4.56)	1,736 / 8,992,476 (1.93)	359 / 1,779,482 (2.02)
**24 Jan-14 Feb**	1,444 / 3,544,088 (4.07)	897 / 1,844,230 (4.86)	503 / 1,588,412 (3.17)	44 / 111,445 (3.95)

medRxiv preprint doi: https://doi.org/10.1101/2021.02.01.21250959; this version posted March 5, 2021. The copyright holder for this preprint
(which was not certified by peer review) is the author/funder, who has granted medRxiv a license to display the preprint in perpetuity.
It is made available under a CC-BY 4.0 International license .

Supplementary Table 2. Cases, deaths, followup days, and deaths per 10,000 days of followup, by SGTF and non-SGTF, cross-tabulated by date range, geographical region, age group, and IMD group. The number in parentheses is the rate of death per 10,000 days of follow-up. EE, LD, SE: East of England, London, South East (regions in which B.1.1.7 was first detected); ML, NEY, NW, SW: Midlands, North East & Yorkshire, North West, South West (other regions).

		**2020-11-01 - 2020-11-20**	
		**EE, LD, SE**	**ML, NEY, NW, SW**
**Age 1 - 54**	IMD 1 - 2	SGTF: 1086 cases, 0 deaths / 30408 days (0)<br>Non-SGTF: 4453 cases, 2 deaths / 124670 days (0.16)	SGTF: 942 cases, 0 deaths / 26376 days (0)<br>Non-SGTF: 44032 cases, 17 deaths / 1232673 days (0.14)
	IMD 3 - 8	SGTF: 4827 cases, 0 deaths / 135156 days (0)<br>Non-SGTF: 24448 cases, 5 deaths / 684449 days (0.07)	SGTF: 1659 cases, 0 deaths / 46452 days (0)<br>Non-SGTF: 68497 cases, 17 deaths / 1917679 days (0.09)
	IMD 9 - 10	SGTF: 1368 cases, 0 deaths / 38304 days (0)<br>Non-SGTF: 7618 cases, 1 deaths / 213286 days (0.05)	SGTF: 369 cases, 0 deaths / 10332 days (0)<br>Non-SGTF: 16586 cases, 4 deaths / 464346 days (0.09)
**Age 55 - 69**	IMD 1 - 2	SGTF: 192 cases, 3 deaths / 5331 days (5.63)<br>Non-SGTF: 599 cases, 7 deaths / 16672 days (4.2)	SGTF: 159 cases, 0 deaths / 4452 days (0)<br>Non-SGTF: 7885 cases, 49 deaths / 220189 days (2.23)
	IMD 3 - 8	SGTF: 800 cases, 4 deaths / 22353 days (1.79)<br>Non-SGTF: 3874 cases, 11 deaths / 108348 days (1.02)	SGTF: 354 cases, 0 deaths / 9912 days (0)<br>Non-SGTF: 15729 cases, 46 deaths / 439885 days (1.05)
	IMD 9 - 10	SGTF: 277 cases, 0 deaths / 7756 days (0)<br>Non-SGTF: 1563 cases, 0 deaths / 43764 days (0)	SGTF: 92 cases, 0 deaths / 2576 days (0)<br>Non-SGTF: 4280 cases, 8 deaths / 119765 days (0.67)
**Age 70 - 84**	IMD 1 - 2	SGTF: 47 cases, 1 deaths / 1296 days (7.72)<br>Non-SGTF: 124 cases, 2 deaths / 3452 days (5.79)	SGTF: 41 cases, 3 deaths / 1112 days (26.98)<br>Non-SGTF: 2195 cases, 97 deaths / 59987.5 days (16.17)
	IMD 3 - 8	SGTF: 166 cases, 4 deaths / 4591 days (8.71)<br>Non-SGTF: 1057 cases, 16 deaths / 29358 days (5.45)	SGTF: 117 cases, 3 deaths / 3226 days (9.3)<br>Non-SGTF: 4402 cases, 143 deaths / 121187 days (11.8)
	IMD 9 - 10	SGTF: 64 cases, 0 deaths / 1792 days (0)<br>Non-SGTF: 436 cases, 8 deaths / 12067 days (6.63)	SGTF: 26 cases, 1 deaths / 722 days (13.85)<br>Non-SGTF: 1225 cases, 38 deaths / 33783 days (11.25)
**Age 85 - 120**	IMD 1 - 2	SGTF: 3 cases, 1 deaths / 83 days (120.48)<br>Non-SGTF: 24 cases, 1 deaths / 658 days (15.2)	SGTF: 4 cases, 0 deaths / 112 days (0)<br>Non-SGTF: 360 cases, 61 deaths / 9140 days (66.74)
	IMD 3 - 8	SGTF: 11 cases, 3 deaths / 276 days (108.7)<br>Non-SGTF: 136 cases, 12 deaths / 3621 days (33.14)	SGTF: 16 cases, 1 deaths / 433 days (23.09)<br>Non-SGTF: 711 cases, 96 deaths / 18480.5 days (51.95)
	IMD 9 - 10	SGTF: 6 cases, 1 deaths / 157 days (63.69)<br>Non-SGTF: 76 cases, 13 deaths / 1956 days (66.46)	SGTF: 5 cases, 1 deaths / 125 days (80)<br>Non-SGTF: 211 cases, 29 deaths / 5455 days (53.16)
		**2020-11-22 - 2020-12-11**	
		**EE, LD, SE**	**ML, NEY, NW, SW**
**Age 1 - 54**	IMD 1 - 2	SGTF: 3850 cases, 1 deaths / 107787 days (0.09)<br>Non-SGTF: 3119 cases, 2 deaths / 87300 days (0.23)	SGTF: 1470 cases, 0 deaths / 41160 days (0)<br>Non-SGTF: 21373 cases, 11 deaths / 598239.5 days (0.18)
	IMD 3 - 8	SGTF: 19213 cases, 10 deaths / 537848 days (0.19)<br>Non-SGTF: 15504 cases, 4 deaths / 434052 days (0.09)	SGTF: 2844 cases, 0 deaths / 79632 days (0)<br>Non-SGTF: 31351 cases, 5 deaths / 877736 days (0.06)
	IMD 9 - 10	SGTF: 5945 cases, 0 deaths / 166460 days (0)<br>Non-SGTF: 4113 cases, 0 deaths / 115164 days (0)	SGTF: 769 cases, 0 deaths / 21532 days (0)<br>Non-SGTF: 7831 cases, 2 deaths / 219241 days (0.09)
**Age 55 - 69**	IMD 1 - 2	SGTF: 474 cases, 3 deaths / 13237 days (2.27)<br>Non-SGTF: 379 cases, 3 deaths / 10591 days (2.83)	SGTF: 176 cases, 1 deaths / 4904 days (2.04)<br>Non-SGTF: 3060 cases, 35 deaths / 85105 days (4.11)
	IMD 3 - 8	SGTF: 2361 cases, 19 deaths / 65965 days (2.88)<br>Non-SGTF: 2023 cases, 9 deaths / 56548 days (1.59)	SGTF: 401 cases, 1 deaths / 11220 days (0.89)<br>Non-SGTF: 6093 cases, 28 deaths / 170247 days (1.64)
	IMD 9 - 10	SGTF: 821 cases, 2 deaths / 22972 days (0.87)<br>Non-SGTF: 689 cases, 2 deaths / 19261 days (1.04)	SGTF: 116 cases, 1 deaths / 3240 days (3.09)<br>Non-SGTF: 1608 cases, 12 deaths / 44909 days (2.67)
**Age 70 - 84**	IMD 1 - 2	SGTF: 75 cases, 5 deaths / 2054 days (24.34)<br>Non-SGTF: 72 cases, 4 deaths / 1926 days (20.77)	SGTF: 37 cases, 4 deaths / 997 days (40.12)<br>Non-SGTF: 821 cases, 37 deaths / 22445 days (16.48)
	IMD 3 - 8	SGTF: 437 cases, 20 deaths / 11963 days (16.72)<br>Non-SGTF: 483 cases, 12 deaths / 13356 days (8.98)	SGTF: 82 cases, 6 deaths / 2198 days (27.3)<br>Non-SGTF: 1689 cases, 67 deaths / 46243 days (14.49)
	IMD 9 - 10	SGTF: 152 cases, 7 deaths / 4144 days (16.89)<br>Non-SGTF: 155 cases, 8 deaths / 4181 days (19.13)	SGTF: 32 cases, 0 deaths / 896 days (0)<br>Non-SGTF: 412 cases, 16 deaths / 11275 days (14.19)
**Age 85 - 120**	IMD 1 - 2	SGTF: 29 cases, 10 deaths / 686 days (145.77)<br>Non-SGTF: 9 cases, 2 deaths / 218 days (91.74)	SGTF: 12 cases, 0 deaths / 336 days (0)<br>Non-SGTF: 206 cases, 32 deaths / 5233 days (61.15)
	IMD 3 - 8	SGTF: 68 cases, 8 deaths / 1788 days (44.74)<br>Non-SGTF: 86 cases, 8 deaths / 2251 days (35.54)	SGTF: 22 cases, 7 deaths / 476.5 days (146.9)<br>Non-SGTF: 411 cases, 63 deaths / 10512 days (59.93)
	IMD 9 - 10	SGTF: 28 cases, 9 deaths / 643 days (139.97)<br>Non-SGTF: 35 cases, 6 deaths / 898 days (66.82)	SGTF: 4 cases, 0 deaths / 112 days (0)<br>Non-SGTF: 116 cases, 14 deaths / 2972 days (47.11)

Continued on next page.

medRxiv preprint doi: https://doi.org/10.1101/2021.02.01.21250959; this version posted March 5, 2021. The copyright holder for this preprint (which was not certified by peer review) is the author/funder, who has granted medRxiv a license to display the preprint in perpetuity.
It is made available under a CC-BY 4.0 International license .

Supplementary Table 2, continued.

		2020-12-13 - 2021-01-01	
		**EE, LD, SE**	**ML, NEY, NW, SW**
**Age 1 - 54**	IMD 1 - 2	SGTF: 14181 cases, 6 deaths / 397009 days (0.15)<br>Non-SGTF: 2467 cases, 2 deaths / 69033 days (0.29)	SGTF: 13313 cases, 16 deaths / 372557 days (0.43)<br>Non-SGTF: 15724 cases, 9 deaths / 440126 days (0.2)
	IMD 3 - 8	SGTF: 76713 cases, 33 deaths / 2147551 days (0.15)<br>Non-SGTF: 13440 cases, 2 deaths / 376279 days (0.05)	SGTF: 23994 cases, 16 deaths / 671660 days (0.24)<br>Non-SGTF: 25618 cases, 15 deaths / 717092 days (0.21)
	IMD 9 - 10	SGTF: 23648 cases, 9 deaths / 662060 days (0.14)<br>Non-SGTF: 3930 cases, 0 deaths / 110040 days (0)	SGTF: 7425 cases, 1 deaths / 207886 days (0.05)<br>Non-SGTF: 6527 cases, 0 deaths / 182756 days (0)
**Age 55 - 69**	IMD 1 - 2	SGTF: 2030 cases, 27 deaths / 56481 days (4.78)<br>Non-SGTF: 361 cases, 1 deaths / 10106 days (0.99)	SGTF: 1949 cases, 30 deaths / 54217 days (5.53)<br>Non-SGTF: 2649 cases, 24 deaths / 73884 days (3.25)
	IMD 3 - 8	SGTF: 12258 cases, 96 deaths / 342029 days (2.81)<br>Non-SGTF: 2177 cases, 10 deaths / 60854 days (1.64)	SGTF: 4926 cases, 39 deaths / 137545 days (2.84)<br>Non-SGTF: 5931 cases, 19 deaths / 165820 days (1.15)
	IMD 9 - 10	SGTF: 4717 cases, 23 deaths / 131754 days (1.75)<br>Non-SGTF: 846 cases, 1 deaths / 23680 days (0.42)	SGTF: 1692 cases, 6 deaths / 47331 days (1.27)<br>Non-SGTF: 1630 cases, 3 deaths / 45599 days (0.66)
**Age 70 - 84**	IMD 1 - 2	SGTF: 349 cases, 21 deaths / 9454 days (22.21)<br>Non-SGTF: 58 cases, 1 deaths / 1610 days (6.21)	SGTF: 443 cases, 28 deaths / 12013 days (23.31)<br>Non-SGTF: 708 cases, 40 deaths / 19256 days (20.77)
	IMD 3 - 8	SGTF: 2601 cases, 99 deaths / 71448 days (13.86)<br>Non-SGTF: 499 cases, 11 deaths / 13811 days (7.96)	SGTF: 1255 cases, 68 deaths / 34198 days (19.88)<br>Non-SGTF: 1722 cases, 68 deaths / 47165 days (14.42)
	IMD 9 - 10	SGTF: 1047 cases, 29 deaths / 28936 days (10.02)<br>Non-SGTF: 187 cases, 6 deaths / 5145 days (11.66)	SGTF: 449 cases, 19 deaths / 12344 days (15.39)<br>Non-SGTF: 437 cases, 11 deaths / 12104 days (9.09)
**Age 85 - 120**	IMD 1 - 2	SGTF: 52 cases, 12 deaths / 1298 days (92.45)<br>Non-SGTF: 3 cases, 2 deaths / 63 days (317.46)	SGTF: 63 cases, 10 deaths / 1607 days (62.23)<br>Non-SGTF: 145 cases, 21 deaths / 3721 days (56.44)
	IMD 3 - 8	SGTF: 284 cases, 39 deaths / 7322 days (53.26)<br>Non-SGTF: 65 cases, 6 deaths / 1696 days (35.38)	SGTF: 180 cases, 37 deaths / 4493 days (82.35)<br>Non-SGTF: 367 cases, 66 deaths / 9204 days (71.71)
	IMD 9 - 10	SGTF: 153 cases, 22 deaths / 3938.5 days (55.86)<br>Non-SGTF: 43 cases, 11 deaths / 987.5 days (111.39)	SGTF: 47 cases, 7 deaths / 1198 days (58.43)<br>Non-SGTF: 92 cases, 15 deaths / 2307 days (65.02)
		**2021-01-03 - 2021-01-22**	
		**EE, LD, SE**	**ML, NEY, NW, SW**
**Age 1 - 54**	IMD 1 - 2	SGTF: 12871 cases, 6 deaths / 354017 days (0.17)<br>Non-SGTF: 696 cases, 1 deaths / 19256 days (0.52)	SGTF: 44708 cases, 48 deaths / 1222863 days (0.39)<br>Non-SGTF: 12412 cases, 5 deaths / 343288 days (0.15)
	IMD 3 - 8	SGTF: 61982 cases, 40 deaths / 1702497 days (0.23)<br>Non-SGTF: 3773 cases, 1 deaths / 104360 days (0.1)	SGTF: 62155 cases, 39 deaths / 1701476 days (0.23)<br>Non-SGTF: 17137 cases, 12 deaths / 473824 days (0.25)
	IMD 9 - 10	SGTF: 14006 cases, 10 deaths / 384876.5 days (0.26)<br>Non-SGTF: 941 cases, 1 deaths / 26011 days (0.38)	SGTF: 14185 cases, 8 deaths / 388859 days (0.21)<br>Non-SGTF: 3510 cases, 3 deaths / 97069 days (0.31)
**Age 55 - 69**	IMD 1 - 2	SGTF: 2024 cases, 18 deaths / 55339 days (3.25)<br>Non-SGTF: 113 cases, 2 deaths / 3105 days (6.44)	SGTF: 7758 cases, 129 deaths / 210715 days (6.12)<br>Non-SGTF: 2368 cases, 15 deaths / 65117 days (2.3)
	IMD 3 - 8	SGTF: 11460 cases, 79 deaths / 313889 days (2.52)<br>Non-SGTF: 646 cases, 4 deaths / 17831 days (2.24)	SGTF: 14056 cases, 123 deaths / 382714 days (3.21)<br>Non-SGTF: 4280 cases, 26 deaths / 117914 days (2.2)
	IMD 9 - 10	SGTF: 3464 cases, 23 deaths / 95017 days (2.42)<br>Non-SGTF: 218 cases, 0 deaths / 5997 days (0)	SGTF: 3787 cases, 22 deaths / 103572 days (2.12)<br>Non-SGTF: 982 cases, 7 deaths / 27089 days (2.58)
**Age 70 - 84**	IMD 1 - 2	SGTF: 387 cases, 11 deaths / 10546 days (10.43)<br>Non-SGTF: 29 cases, 2 deaths / 767 days (26.08)	SGTF: 1941 cases, 152 deaths / 50738 days (29.96)<br>Non-SGTF: 702 cases, 56 deaths / 18460.5 days (30.34)
	IMD 3 - 8	SGTF: 2246 cases, 95 deaths / 60410.5 days (15.73)<br>Non-SGTF: 156 cases, 1 deaths / 4279 days (2.34)	SGTF: 3779 cases, 242 deaths / 99990 days (24.2)<br>Non-SGTF: 1371 cases, 56 deaths / 36970.5 days (15.15)
	IMD 9 - 10	SGTF: 817 cases, 32 deaths / 21875 days (14.63)<br>Non-SGTF: 58 cases, 4 deaths / 1546 days (25.87)	SGTF: 1009 cases, 41 deaths / 27043 days (15.16)<br>Non-SGTF: 365 cases, 14 deaths / 9867 days (14.19)
**Age 85 - 120**	IMD 1 - 2	SGTF: 50 cases, 8 deaths / 1225 days (65.31)<br>Non-SGTF: 5 cases, 1 deaths / 111 days (90.09)	SGTF: 427 cases, 97 deaths / 10060 days (96.42)<br>Non-SGTF: 215 cases, 47 deaths / 5074 days (92.63)
	IMD 3 - 8	SGTF: 431 cases, 63 deaths / 10772 days (58.48)<br>Non-SGTF: 33 cases, 7 deaths / 792 days (88.38)	SGTF: 985 cases, 201 deaths / 23329.5 days (86.16)<br>Non-SGTF: 388 cases, 60 deaths / 9692 days (61.91)
	IMD 9 - 10	SGTF: 229 cases, 46 deaths / 5580 days (82.44)<br>Non-SGTF: 19 cases, 1 deaths / 512 days (19.53)	SGTF: 266 cases, 59 deaths / 6271 days (94.08)<br>Non-SGTF: 100 cases, 13 deaths / 2584 days (50.31)
		**2021-01-24 - 2021-02-14**	
		**EE, LD, SE**	**ML, NEY, NW, SW**
**Age 1 - 54**	IMD 1 - 2	SGTF: 3888 cases, 2 deaths / 52209 days (0.38)<br>Non-SGTF: 114 cases, 0 deaths / 1656.5 days (0)	SGTF: 27972 cases, 13 deaths / 326729.5 days (0.4)<br>Non-SGTF: 1846 cases, 1 deaths / 26474.5 days (0.38)
	IMD 3 - 8	SGTF: 20931 cases, 4 deaths / 278769 days (0.14)<br>Non-SGTF: 584 cases, 0 deaths / 8290.5 days (0)	SGTF: 36411 cases, 9 deaths / 432872 days (0.21)<br>Non-SGTF: 2458 cases, 1 deaths / 35407 days (0.28)
	IMD 9 - 10	SGTF: 5353 cases, 0 deaths / 69844.5 days (0)<br>Non-SGTF: 161 cases, 0 deaths / 2380.5 days (0)	SGTF: 7623 cases, 1 deaths / 91848 days (0.11)<br>Non-SGTF: 498 cases, 0 deaths / 7288.5 days (0)
**Age 55 - 69**	IMD 1 - 2	SGTF: 590 cases, 5 deaths / 8061 days (6.2)<br>Non-SGTF: 17 cases, 0 deaths / 223 days (0)	SGTF: 4792 cases, 27 deaths / 56571 days (4.77)<br>Non-SGTF: 401 cases, 2 deaths / 5922.5 days (3.38)
	IMD 3 - 8	SGTF: 3753 cases, 15 deaths / 51132.5 days (2.93)<br>Non-SGTF: 113 cases, 2 deaths / 1683 days (11.88)	SGTF: 8245 cases, 45 deaths / 99120 days (4.54)<br>Non-SGTF: 655 cases, 3 deaths / 9578.5 days (3.13)
	IMD 9 - 10	SGTF: 1329 cases, 3 deaths / 17341.5 days (1.73)<br>Non-SGTF: 62 cases, 0 deaths / 891 days (0)	SGTF: 1993 cases, 2 deaths / 23714.5 days (0.84)<br>Non-SGTF: 172 cases, 0 deaths / 2462 days (0)
**Age 70 - 84**	IMD 1 - 2	SGTF: 109 cases, 1 deaths / 1557 days (6.42)<br>Non-SGTF: 0 cases, 0 deaths / 0 days (NaN)	SGTF: 1170 cases, 44 deaths / 14501 days (30.34)<br>Non-SGTF: 118 cases, 6 deaths / 1611 days (37.24)
	IMD 3 - 8	SGTF: 770 cases, 26 deaths / 10242.5 days (25.38)<br>Non-SGTF: 27 cases, 0 deaths / 415 days (0)	SGTF: 2006 cases, 82 deaths / 24108 days (34.01)<br>Non-SGTF: 256 cases, 6 deaths / 3509.5 days (17.1)
	IMD 9 - 10	SGTF: 313 cases, 11 deaths / 4040.5 days (27.22)<br>Non-SGTF: 10 cases, 0 deaths / 148 days (0)	SGTF: 477 cases, 19 deaths / 5939 days (31.99)<br>Non-SGTF: 58 cases, 2 deaths / 755.5 days (26.47)
**Age 85 - 120**	IMD 1 - 2	SGTF: 11 cases, 2 deaths / 167 days (119.76)<br>Non-SGTF: 0 cases, 0 deaths / 0 days (NaN)	SGTF: 357 cases, 42 deaths / 4102.5 days (102.38)<br>Non-SGTF: 54 cases, 2 deaths / 848 days (23.58)
	IMD 3 - 8	SGTF: 198 cases, 21 deaths / 2614 days (80.34)<br>Non-SGTF: 10 cases, 2 deaths / 114 days (175.44)	SGTF: 769 cases, 93 deaths / 9061.5 days (102.63)<br>Non-SGTF: 119 cases, 13 deaths / 1528.5 days (85.05)
	IMD 9 - 10	SGTF: 112 cases, 10 deaths / 1409 days (70.97)<br>Non-SGTF: 3 cases, 0 deaths / 41 days (0)	SGTF: 207 cases, 26 deaths / 2458 days (105.78)<br>Non-SGTF: 15 cases, 4 deaths / 217 days (184.33)

medRxiv preprint doi: https://doi.org/10.1101/2021.02.01.21250959; this version posted March 5, 2021. The copyright holder for this preprint (which was not certified by peer review) is the author/funder, who has granted medRxiv a license to display the preprint in perpetuity.
It is made available under a CC-BY 4.0 International license .

Supplementary Note 1
Models with interaction terms

In the main text (section "Cox regression analyses"), we briefly describe results obtained from models including interactions between SGTF and other covariates, as well as interactions between other covariates and time since positive test, in our complete-cases analysis.

As stated in the main text, in our analysis of the effect of SGTF on the hazard of death due to COVID-19, we found no evidence that the effect of SGTF varied by age group, sex, IMD, ethnicity, or residence type. In an earlier analysis using data up to 25 January 2021, we did find a marginally significant interaction (likelihood ratio test $P(\chi^2_2 = 6.8) = 0.034$) between SGTF and residence type indicating a higher hazard of death due to SGTF in care/nursing home residents, but this interaction is no longer present (likelihood ratio test $P(\chi^2_2 = 0.33) = 0.85$) when analysing the more complete data set up to 14 February 2021.

We also describe in the main text a significant interaction between SGTF and time since positive test (henceforth, "time since positive test" is abbreviated as "time", not to be confused with the date upon which the specimen was taken). In additional analyses, we also found significant interactions between age and time, sex and time, and place of residence and time.

Specifically, in our complete-cases analysis:
- the SGTF:time coefficient was estimated at 0.025 (standard error 0.0076), indicating a slightly longer time from specimen to death for individuals with SGTF (likelihood ratio test $P(\chi^2_1 = 11) = 0.0009$);
- the age:time coefficient was estimated at $-8.3\text{x}10^{-4}$ (SE $1.9\text{x}10^{-4}$), indicating a slightly shorter time from specimen to death for older individuals (likelihood ratio test $P(\chi^2_1 = 20) = 9 \times 10^{-6}$);
- the sex_{male}:time coefficient was estimated at $9.4\text{x}10^{-3}$ (SE $4.7\text{x}10^{-3}$), indicating a slightly longer time from specimen to death for males (likelihood ratio test $P(\chi^2_1 = 4.0) = 0.046$); and
- the $\text{residence}_{\text{care/nursing home}}$:time coefficient was estimated at –0.024 (SE 0.010), indicating a slightly shorter time from specimen to death for care home residents (likelihood ratio test $P(\chi^2_2 = 7.5) = 0.023$).

By contrast, we did not find any significant interaction between ethnicity and time (likelihood ratio test $P(\chi^2_3 = 0.48) = 0.92$) or between IMD decile and time (likelihood ratio test $P(\chi^2_1 = 0.0042) = 0.95$).

medRxiv preprint doi: https://doi.org/10.1101/2021.02.01.21250959; this version posted March 5, 2021. The copyright holder for this preprint (which was not certified by peer review) is the author/funder, who has granted medRxiv a license to display the preprint in perpetuity.
It is made available under a CC-BY 4.0 International license .

Supplementary Note 2
CMMID COVID-19 Working Group acknowledgements

Funding statements for the CMMID COVID-19 working group are as follows. KvZ: KvZ is supported by the UK Foreign, Commonwealth and Development Office (FCDO)/Wellcome Trust Epidemic Preparedness Coronavirus research programme (ref. 221303/Z/20/Z), and Elrha's Research for Health in Humanitarian Crises (R2HC) Programme, which aims to improve health outcomes by strengthening the evidence base for public health interventions in humanitarian crises. The R2HC programme is funded by the UK Government (FCDO), the Wellcome Trust, and the UK National Institute for Health Research (NIHR). SC: Wellcome Trust (grant: 208812/Z/17/Z). FYS: NIHR EPIC grant (16/137/109). SFunk: Wellcome Trust (grant: 210758/Z/18/Z), NIHR (NIHR200908). GFM: NTD Modelling Consortium by the Bill and Melinda Gates Foundation (OPP1184344). YJ: LSHTM, DHSC/UKRI COVID-19 Rapid Response Initiative. SRM: Wellcome Trust (grant: 210758/Z/18/Z). RL: Royal Society Dorothy Hodgkin Fellowship. WJE: European Commission (EpiPose 101003688), NIHR (NIHR200908). MQ: European Research Council Starting Grant (Action Number #757699); Bill and Melinda Gates Foundation (INV-001754). NRW: Medical Research Council (grant number MR/N013638/1). RME: HDR UK (grant: MR/S003975/1), MRC (grant: MC_PC 19065), NIHR (grant: NIHR200908). NGD: UKRI Research England; NIHR Health Protection Research Unit in Immunisation (NIHR200929); UK MRC (MC_PC_19065). JYL: Bill & Melinda Gates Foundation (INV-003174). MK: Foreign, Commonwealth and Development Office / Wellcome Trust. FK: Innovation Fund of the Joint Federal Committee (Grant number 01VSF18015), Wellcome Trust (UNS110424). DCT: No funding declared. JDM: Wellcome Trust (grant: 210758/Z/18/Z). AS: No funding declared. AMF: No funding declared. KP: Gates (INV-003174), European Commission (101003688). SFlasche: Wellcome Trust (grant: 208812/Z/17/Z). AJK: Wellcome Trust (grant: 206250/Z/17/Z), NIHR (NIHR200908). SA: Wellcome Trust (grant: 210758/Z/18/Z). BJQ: This research was partly funded by the National Institute for Health Research (NIHR) (16/137/109 & 16/136/46) using UK aid from the UK Government to support global health research. The views expressed in this publication are those of the author(s) and not necessarily those of the NIHR or the UK Department of Health and Social Care. BJQ is supported in part by a grant from the Bill and Melinda Gates Foundation (OPP1139859). TJ: RCUK/ESRC (grant: ES/P010873/1); UK PH RST; NIHR HPRU Modelling & Health Economics (NIHR200908). AR: NIHR (grant: PR-OD-1017-20002). GMK: UK Medical Research Council (grant: MR/P014658/1). MJ: Gates (INV-003174, INV-016832), NIHR (16/137/109, NIHR200929, NIHR200908), European Commission (EpiPose 101003688). YL: Gates (INV-003174), NIHR (16/137/109), European Commission (101003688). JW: NIHR Health Protection Research Unit and NIHR HTA. JH: Wellcome Trust (grant: 210758/Z/18/Z). KO'R: Bill and Melinda Gates Foundation (OPP1191821). YWDC: No funding declared. TWR: Wellcome Trust (grant: 206250/Z/17/Z). CIJ: Global Challenges Research Fund (GCRF) project 'RECAP' managed through RCUK and ESRC (ES/P010873/1). SRP: Bill and Melinda Gates Foundation (INV-016832). AE: The Nakajima Foundation. ESN: Gates (OPP1183986). NIB: Health Protection Research Unit (grant code NIHR200908). CJVA: European Research Council Starting Grant (Action number 757688). FGS: NIHR Health Protection Research Unit in Modelling & Health Economics, and in Immunisation. AG: European Commission (EpiPose 101003688). KA: Bill & Melinda Gates Foundation (OPP1157270, INV-

medRxiv preprint doi: https://doi.org/10.1101/2021.02.01.21250959; this version posted March 5, 2021. The copyright holder for this preprint (which was not certified by peer review) is the author/funder, who has granted medRxiv a license to display the preprint in perpetuity.
It is made available under a CC-BY 4.0 International license .

016832). WW: MRC (grant MR/V027956/1). KEA: European Research Council Starting Grant (Action number 757688). RCB: European Commission (EpiPose 101003688). PK: This research was partly funded by the Royal Society under award RP\EA\180004, European Commission (101003688), Bill & Melinda Gates Foundation (INV-003174). HPG: This research was produced by CSIGN which is part of the EDCTP2 programme supported by the European Union (grant number RIA2020EF-2983-CSIGN). The views and opinions of authors expressed herein do not necessarily state or reflect those of EDCTP. This research is funded by the Department of Health and Social Care using UK Aid funding and is managed by the NIHR. The views expressed in this publication are those of the author(s) and not necessarily those of the Department of Health and Social Care (PR-OD-1017-20001). CABP: CABP is supported by the Bill & Melinda Gates Foundation (OPP1184344) and the UK Foreign, Commonwealth and Development Office (FCDO)/Wellcome Trust Epidemic Preparedness Coronavirus research programme (ref. 221303/Z/20/Z). OJB: Wellcome Trust (grant: 206471/Z/17/Z).
