# Supplementary material for: Increased hazard of death in community-tested cases of SARS-CoV-2 Variant of Concern 202012/01
Source: medRxiv. 2021 Feb 3:2021.02.01.21250959. Preprint. [Version 1] doi: 10.1101/2021.02.01.21250959 (PMC7872389; doi:10.1101/2021.02.01.21250959)
Supplement: Supplementary file 1 [file NIHPP2021.02.01.21250959-supplement-1.pdf]

medRxiv preprint doi: https://doi.org/10.1101/2021.02.01.21250959; this version posted February 3, 2021. The copyright holder for this preprint (which was not certified by peer review) is the author/funder, who has granted medRxiv a license to display the preprint in perpetuity.
It is made available under a CC-BY 4.0 International license .

Supplementary tables

Table S1. Characteristics of study subjects, including missing SGTF status. Specimens from 1 Nov 2020 to 11 January 2021.

	All	Missing	SGTF	Non-SGTF
	1,625,121 (100%)	766,940 (100%)	411,961 (100%)	446,220 (100%)
**Sex**				
**Female**	874,833 (53.8%)	425,145 (55.4%)	213,374 (51.8%)	236,314 (53%)
**Male**	750,288 (46.2%)	341,795 (44.6%)	198,587 (48.2%)	209,906 (47%)
**Age**				
**1–34**	729,865 (44.9%)	340,711 (44.4%)	188,967 (45.9%)	200,187 (44.9%)
**35–54**	556,849 (34.3%)	254,889 (33.2%)	147,747 (35.9%)	154,213 (34.6%)
**55–69**	240,984 (14.8%)	112,140 (14.6%)	59,664 (14.5%)	69,180 (15.5%)
**70–84**	69,560 (4.3%)	37,088 (4.8%)	13,495 (3.3%)	18,977 (4.3%)
**85 and older**	27,863 (1.7%)	22,112 (2.9%)	2,088 (0.5%)	3,663 (0.8%)
**Place of residence**				
**Residential**	1,518,839 (93.5%)	693,959 (90.5%)	397,203 (96.4%)	427,677 (95.8%)
**Care/Nursing home**	47,104 (2.9%)	42,435 (5.5%)	1,435 (0.3%)	3,234 (0.7%)
**Other/Unknown**	59,178 (3.6%)	30,546 (4%)	13,323 (3.2%)	15,309 (3.4%)
**Ethnicity**				
**White**	1,202,741 (74%)	564,221 (73.6%)	297,863 (72.3%)	340,657 (76.3%)
**Asian**	223,071 (13.7%)	97,395 (12.7%)	59,512 (14.4%)	66,164 (14.8%)
**Black**	73,628 (4.5%)	39,780 (5.2%)	21,028 (5.1%)	12,820 (2.9%)
**Other/Mixed/Unknown**	125,681 (7.7%)	65,544 (8.5%)	33,558 (8.1%)	26,579 (6%)
**Index of Multiple Deprivation decile**				
**1**	162,761 (10%)	57,550 (7.5%)	35,913 (8.7%)	69,298 (15.5%)
**2**	190,790 (11.7%)	87,541 (11.4%)	46,668 (11.3%)	56,581 (12.7%)
**3**	194,098 (11.9%)	94,080 (12.3%)	48,777 (11.8%)	51,241 (11.5%)
**4**	178,493 (11%)	87,934 (11.5%)	45,849 (11.1%)	44,710 (10%)

medRxiv preprint doi: https://doi.org/10.1101/2021.02.01.21250959; this version posted February 3, 2021. The copyright holder for this preprint (which was not certified by peer review) is the author/funder, who has granted medRxiv a license to display the preprint in perpetuity.
It is made available under a CC-BY 4.0 International license .

**5**	167,919 (10.3%)	83,117 (10.8%)	43,639 (10.6%)	41,163 (9.2%)
**6**	158,095 (9.7%)	79,334 (10.3%)	40,317 (9.8%)	38,444 (8.6%)
**7**	151,551 (9.3%)	74,184 (9.7%)	38,565 (9.4%)	38,802 (8.7%)
**8**	148,871 (9.2%)	71,854 (9.4%)	38,050 (9.2%)	38,967 (8.7%)
**9**	144,127 (8.9%)	69,822 (9.1%)	38,257 (9.3%)	36,048 (8.1%)
**10**	128,416 (7.9%)	61,524 (8%)	35,926 (8.7%)	30,966 (6.9%)
**NHS England region**				
**East of England**	205,900 (12.7%)	130,286 (17%)	54,339 (13.2%)	21,275 (4.8%)
**London**	388,515 (23.9%)	222,400 (29%)	117,899 (28.6%)	48,216 (10.8%)
**Midlands**	287,007 (17.7%)	117,096 (15.3%)	61,254 (14.9%)	108,657 (24.4%)
**North East and Yorkshire**	203,332 (12.5%)	45,367 (5.9%)	37,400 (9.1%)	120,565 (27%)
**North West**	182,175 (11.2%)	42,266 (5.5%)	43,112 (10.5%)	96,797 (21.7%)
**South East**	266,809 (16.4%)	146,097 (19%)	86,656 (21%)	34,056 (7.6%)
**South West**	91,383 (5.6%)	63,428 (8.3%)	11,301 (2.7%)	16,654 (3.7%)
**Specimen date**				
**1 Nov–14 Nov**	251,518 (15.5%)	87,023 (11.3%)	8,029 (1.9%)	156,466 (35.1%)
**15 Nov–28 Nov**	168,921 (10.4%)	57,723 (7.5%)	12,243 (3%)	98,955 (22.2%)
**29 Nov–12 Dec**	166,505 (10.2%)	61,640 (8%)	37,910 (9.2%)	66,955 (15%)
**13 Dec–26 Dec**	356,566 (21.9%)	187,075 (24.4%)	111,367 (27%)	58,124 (13%)
**27 Dec–11 Jan**	681,611 (41.9%)	373,479 (48.7%)	242,412 (58.8%)	65,720 (14.7%)

medRxiv preprint doi: https://doi.org/10.1101/2021.02.01.21250959; this version posted February 3, 2021. The copyright holder for this preprint (which was not certified by peer review) is the author/funder, who has granted medRxiv a license to display the preprint in perpetuity.
It is made available under a CC-BY 4.0 International license .

Table S2. Deaths within 28 days of a positive test among study subjects, including missing SGTF status

	All	Missing	SGTF	Non-SGTF
	7,459 (100%)	5,368 (100%)	687 (100%)	1,404 (100%)
**Sex**				
**Female**	3,909 (52.4%)	3,014 (56.1%)	283 (41.2%)	612 (43.6%)
**Male**	3,550 (47.6%)	2,354 (43.9%)	404 (58.8%)	792 (56.4%)
**Age**				
**1–34**	32 (0.4%)	13 (0.2%)	6 (0.9%)	13 (0.9%)
**35–54**	272 (3.6%)	131 (2.4%)	57 (8.3%)	84 (6%)
**55–69**	814 (10.9%)	379 (7.1%)	173 (25.2%)	262 (18.7%)
**70–84**	2,566 (34.4%)	1,697 (31.6%)	287 (41.8%)	582 (41.5%)
**85 and older**	3,775 (50.6%)	3,148 (58.6%)	164 (23.9%)	463 (33%)
**Place of residence**				
**Residential**	2,702 (36.2%)	1,133 (21.1%)	547 (79.6%)	1,022 (72.8%)
**Care/Nursing home**	4,555 (61.1%)	4,080 (76%)	123 (17.9%)	352 (25.1%)
**Other/Unknown**	202 (2.7%)	155 (2.9%)	17 (2.5%)	30 (2.1%)
**Ethnicity**				
**White**	6,652 (89.2%)	4,947 (92.2%)	532 (77.4%)	1,173 (83.5%)
**Asian**	522 (7%)	244 (4.5%)	109 (15.9%)	169 (12%)
**Black**	116 (1.6%)	62 (1.2%)	28 (4.1%)	26 (1.9%)
**Other/Mixed/Unknown**	169 (2.3%)	115 (2.1%)	18 (2.6%)	36 (2.6%)
**Index of Multiple Deprivation decile**				
**1**	789 (10.6%)	448 (8.3%)	72 (10.5%)	269 (19.2%)
**2**	813 (10.9%)	532 (9.9%)	88 (12.8%)	193 (13.7%)
**3**	769 (10.3%)	544 (10.1%)	53 (7.7%)	172 (12.3%)
**4**	787 (10.6%)	581 (10.8%)	81 (11.8%)	125 (8.9%)

medRxiv preprint doi: https://doi.org/10.1101/2021.02.01.21250959; this version posted February 3, 2021. The copyright holder for this preprint (which was not certified by peer review) is the author/funder, who has granted medRxiv a license to display the preprint in perpetuity.
It is made available under a CC-BY 4.0 International license .

**5**	753 (10.1%)	577 (10.7%)	70 (10.2%)	106 (7.5%)
**6**	741 (9.9%)	545 (10.2%)	81 (11.8%)	115 (8.2%)
**7**	752 (10.1%)	595 (11.1%)	61 (8.9%)	96 (6.8%)
**8**	711 (9.5%)	527 (9.8%)	73 (10.6%)	111 (7.9%)
**9**	747 (10%)	576 (10.7%)	56 (8.2%)	115 (8.2%)
**10**	597 (8%)	443 (8.3%)	52 (7.6%)	102 (7.3%)
**NHS England region**				
**East of England**	848 (11.4%)	697 (13%)	85 (12.4%)	66 (4.7%)
**London**	744 (10%)	515 (9.6%)	162 (23.6%)	67 (4.8%)
**Midlands**	1,688 (22.6%)	1,243 (23.2%)	80 (11.6%)	365 (26%)
**North East and Yorkshire**	1,351 (18.1%)	748 (13.9%)	91 (13.2%)	512 (36.5%)
**North West**	797 (10.7%)	464 (8.6%)	64 (9.3%)	269 (19.2%)
**South East**	1,443 (19.3%)	1,195 (22.3%)	187 (27.2%)	61 (4.3%)
**South West**	588 (7.9%)	506 (9.4%)	18 (2.6%)	64 (4.6%)
**Specimen date**				
**1 Nov–14 Nov**	1,463 (19.6%)	915 (17%)	20 (2.9%)	528 (37.6%)
**15 Nov–28 Nov**	1,261 (16.9%)	922 (17.2%)	26 (3.8%)	313 (22.3%)
**29 Nov–12 Dec**	1,406 (18.8%)	1,016 (18.9%)	124 (18%)	266 (18.9%)
**13 Dec–26 Dec**	1,838 (24.6%)	1,363 (25.4%)	281 (40.9%)	194 (13.8%)
**27 Dec–11 Jan**	1,491 (20%)	1,152 (21.5%)	236 (34.4%)	103 (7.3%)

526

medRxiv preprint doi: https://doi.org/10.1101/2021.02.01.21250959; this version posted February 3, 2021. The copyright holder for this preprint (which was not certified by peer review) is the author/funder, who has granted medRxiv a license to display the preprint in perpetuity.
It is made available under a CC-BY 4.0 International license .

Table S3. Rates of death within 28 days of positive test among study subjects, including missing SGTF status. Total number of deaths, number of days of followup, and deaths per 10,000 days of followup reported.

	All	Missing	SGTF	Non-SGTF
	7,459 / 29,601,000 (2.52)	5,368 / 12,824,834 (4.19)	687 / 5,882,304 (1.17)	1,404 / 10,893,861 (1.29)
**Sex**				
**Female**	3,909 / 15,961,968 (2.45)	3,014 / 7,138,615 (4.22)	283 / 3,055,682 (0.93)	612 / 5,767,671 (1.06)
**Male**	3,550 / 13,639,032 (2.6)	2,354 / 5,686,220 (4.14)	404 / 2,826,622 (1.43)	792 / 5,126,190 (1.55)
**Age**				
**1–34**	32 / 13,481,680 (0.02)	13 / 5,787,812 (0.02)	6 / 2,767,823 (0.02)	13 / 4,926,044 (0.03)
**35–54**	272 / 10,167,962 (0.27)	131 / 4,260,552 (0.31)	57 / 2,133,536 (0.27)	84 / 3,773,873 (0.22)
**55–69**	814 / 4,287,546 (1.9)	379 / 1,829,014 (2.07)	173 / 789,704 (2.19)	262 / 1,668,828 (1.57)
**70–84**	2,566 / 1,213,482 (21.15)	1,697 / 598,176 (28.37)	287 / 168,523 (17.03)	582 / 446,784 (13.03)
**85 and older**	3,775 / 450,330 (83.83)	3,148 / 349,280 (90.13)	164 / 22,718 (72.19)	463 / 78,332 (59.11)
**Place of residence**				
**Residential**	2,702 / 27,653,420 (0.98)	1,133 / 11,545,350 (0.98)	547 / 5,668,687 (0.96)	1,022 / 10,439,383 (0.98)
**Care/Nursing home**	4,555 / 844,984 (53.91)	4,080 / 757,984 (53.83)	123 / 17,672 (69.6)	352 / 69,329 (50.77)
**Other/Unknown**	202 / 1,102,596 (1.83)	155 / 521,502 (2.97)	17 / 195,946 (0.87)	30 / 385,149 (0.78)
**Ethnicity**				
**White**	6,652 / 22,097,241 (3.01)	4,947 / 9,555,278 (5.18)	532 / 4,275,094 (1.24)	1,173 / 8,266,870 (1.42)
**Asian**	522 / 4,102,698 (1.27)	244 / 1,618,328 (1.51)	109 / 827,494 (1.32)	169 / 1,656,876 (1.02)
**Black**	116 / 1,198,700 (0.97)	62 / 600,677 (1.03)	28 / 286,850 (0.98)	26 / 311,174 (0.84)
**Other/Mixed/Unknown**	169 / 2,202,360 (0.77)	115 / 1,050,551 (1.09)	18 / 492,868 (0.37)	36 / 658,941 (0.55)
**Index of Multiple Deprivation decile**				
**1**	789 / 3,104,612 (2.54)	448 / 1,043,579 (4.29)	72 / 393,858 (1.83)	269 / 1,667,175 (1.61)
**2**	813 / 3,461,175 (2.35)	532 / 1,458,037 (3.65)	88 / 622,894 (1.41)	193 / 1,380,244 (1.4)
**3**	769 / 3,481,612 (2.21)	544 / 1,538,028 (3.54)	53 / 689,400 (0.77)	172 / 1,254,184 (1.37)
**4**	787 / 3,180,420 (2.47)	581 / 1,433,450 (4.05)	81 / 652,995 (1.24)	125 / 1,093,974 (1.14)
**5**	753 / 3,015,647 (2.5)	577 / 1,369,916 (4.21)	70 / 642,930 (1.09)	106 / 1,002,802 (1.06)
**6**	741 / 2,847,643 (2.6)	545 / 1,308,278 (4.17)	81 / 598,790 (1.35)	115 / 940,576 (1.22)

medRxiv preprint doi: https://doi.org/10.1101/2021.02.01.21250959; this version posted February 3, 2021. The copyright holder for this preprint (which was not certified by peer review) is the author/funder, who has granted medRxiv a license to display the preprint in perpetuity.
It is made available under a CC-BY 4.0 International license .

**7**	752 / 2,777,872 (2.71)	595 / 1,249,524 (4.76)	61 / 577,502 (1.06)	96 / 950,846 (1.01)
**8**	711 / 2,726,232 (2.61)	527 / 1,208,752 (4.36)	73 / 565,228 (1.29)	111 / 952,252 (1.17)
**9**	747 / 2,650,394 (2.82)	576 / 1,176,614 (4.9)	56 / 586,332 (0.96)	115 / 887,449 (1.3)
**10**	597 / 2,355,392 (2.53)	443 / 1,038,658 (4.27)	52 / 552,376 (0.94)	102 / 764,360 (1.33)
**NHS England region**				
**East of England**	848 / 3,509,872 (2.42)	697 / 2,120,456 (3.29)	85 / 865,050 (0.98)	66 / 524,366 (1.26)
**London**	744 / 6,636,995 (1.12)	515 / 3,428,873 (1.5)	162 / 1,976,046 (0.82)	67 / 1,232,076 (0.54)
**Midlands**	1,688 / 5,506,792 (3.07)	1,243 / 2,192,040 (5.67)	80 / 635,712 (1.26)	365 / 2,679,039 (1.36)
**North East and Yorkshire**	1,351 / 4,309,108 (3.14)	748 / 915,740 (8.17)	91 / 418,625 (2.17)	512 / 2,974,742 (1.72)
**North West**	797 / 3,350,266 (2.38)	464 / 746,482 (6.22)	64 / 372,149 (1.72)	269 / 2,231,635 (1.21)
**South East**	1,443 / 4,675,060 (3.09)	1,195 / 2,347,994 (5.09)	187 / 1,472,920 (1.27)	61 / 854,146 (0.71)
**South West**	588 / 1,612,909 (3.65)	506 / 1,073,250 (4.71)	18 / 141,804 (1.27)	64 / 397,856 (1.61)
**Specimen date**				
**1 Nov–14 Nov**	1,463 / 7,020,658 (2.08)	915 / 2,422,422 (3.78)	20 / 224,551 (0.89)	528 / 4,373,686 (1.21)
**15 Nov–28 Nov**	1,261 / 4,709,996 (2.68)	922 / 1,601,682 (5.76)	26 / 342,473 (0.76)	313 / 2,765,842 (1.13)
**29 Nov–12 Dec**	1,406 / 4,640,583 (3.03)	1,016 / 1,709,998 (5.94)	124 / 1,059,924 (1.17)	266 / 1,870,662 (1.42)
**13 Dec–26 Dec**	1,838 / 7,889,946 (2.33)	1,363 / 4,083,340 (3.34)	281 / 2,463,718 (1.14)	194 / 1,342,890 (1.44)
**27 Dec–11 Jan**	1,491 / 5,339,816 (2.79)	1,152 / 3,007,394 (3.83)	236 / 1,791,640 (1.32)	103 / 540,782 (1.9)

medRxiv preprint doi: https://doi.org/10.1101/2021.02.01.21250959; this version posted February 3, 2021. The copyright holder for this preprint (which was not certified by peer review) is the author/funder, who has granted medRxiv a license to display the preprint in perpetuity.
It is made available under a CC-BY 4.0 International license .

Table S4. Rates of death within any time period following positive test among study subjects, including missing SGTF status. Total number of deaths, number of days of followup, and deaths per 10,000 days of followup reported.

	All	Missing	SGTF	Non-SGTF
	8,091 / 43,913,229 (1.84)	5,776 / 17,737,170 (3.26)	705 / 6,696,880 (1.05)	1,610 / 19,479,180 (0.83)
**Sex**				
**Female**	4,249 / 23,676,604 (1.79)	3,261 / 9,890,125 (3.3)	290 / 3,481,554 (0.83)	698 / 10,304,925 (0.68)
**Male**	3,842 / 20,236,626 (1.9)	2,515 / 7,847,044 (3.21)	415 / 3,215,326 (1.29)	912 / 9,174,255 (0.99)
**Age**				
**1–34**	36 / 19,921,946 (0.02)	13 / 7,977,194 (0.02)	6 / 3,162,130 (0.02)	17 / 8,782,623 (0.02)
**35–54**	304 / 14,986,480 (0.2)	140 / 5,833,410 (0.24)	60 / 2,421,846 (0.25)	104 / 6,731,224 (0.15)
**55–69**	920 / 6,489,470 (1.42)	410 / 2,570,893 (1.59)	178 / 895,650 (1.99)	332 / 3,022,926 (1.1)
**70–84**	2,773 / 1,855,836 (14.94)	1,831 / 854,882 (21.42)	293 / 191,878 (15.27)	649 / 809,076 (8.02)
**85 and older**	4,058 / 659,498 (61.53)	3,382 / 500,791 (67.53)	168 / 25,376 (66.21)	508 / 133,331 (38.1)
**Place of residence**				
**Residential**	2,965 / 40,917,878 (0.72)	1,207 / 15,811,690 (0.76)	560 / 6,451,174 (0.87)	1,198 / 18,655,015 (0.64)
**Care/Nursing home**	4,916 / 1,331,478 (36.92)	4,408 / 1,190,394 (37.03)	127 / 20,434 (62.15)	381 / 120,649 (31.58)
**Other/Unknown**	210 / 1,663,873 (1.26)	161 / 735,086 (2.19)	18 / 225,272 (0.8)	31 / 703,516 (0.44)
**Ethnicity**				
**White**	7,225 / 33,130,923 (2.18)	5,332 / 13,340,104 (4)	548 / 4,894,532 (1.12)	1,345 / 14,896,288 (0.9)
**Asian**	553 / 6,053,338 (0.91)	250 / 2,220,844 (1.13)	109 / 926,274 (1.18)	194 / 2,906,220 (0.67)
**Black**	126 / 1,635,050 (0.77)	65 / 783,512 (0.83)	30 / 322,224 (0.93)	31 / 529,314 (0.59)
**Other/Mixed/Unknown**	187 / 3,093,917 (0.6)	129 / 1,392,709 (0.93)	18 / 553,850 (0.32)	40 / 1,147,358 (0.35)
**Index of Multiple Deprivation decile**				
**1**	873 / 5,075,570 (1.72)	493 / 1,610,402 (3.06)	73 / 454,148 (1.61)	307 / 3,011,020 (1.02)
**2**	885 / 5,222,354 (1.69)	573 / 2,036,766 (2.81)	90 / 707,200 (1.27)	222 / 2,478,388 (0.9)
**3**	826 / 5,107,348 (1.62)	582 / 2,089,530 (2.79)	53 / 784,698 (0.68)	191 / 2,233,120 (0.86)
**4**	851 / 4,650,239 (1.83)	626 / 1,961,380 (3.19)	84 / 741,152 (1.13)	141 / 1,947,706 (0.72)
**5**	808 / 4,387,421 (1.84)	608 / 1,867,574 (3.26)	72 / 735,742 (0.98)	128 / 1,784,104 (0.72)
**6**	798 / 4,139,651 (1.93)	584 / 1,786,786 (3.27)	82 / 682,912 (1.2)	132 / 1,669,954 (0.79)

medRxiv preprint doi: https://doi.org/10.1101/2021.02.01.21250959; this version posted February 3, 2021. The copyright holder for this preprint (which was not certified by peer review) is the author/funder, who has granted medRxiv a license to display the preprint in perpetuity.
It is made available under a CC-BY 4.0 International license .

**7**	828 / 4,079,280 (2.03)	647 / 1,717,204 (3.77)	62 / 659,002 (0.94)	119 / 1,703,074 (0.7)
**8**	756 / 4,000,498 (1.89)	557 / 1,652,206 (3.37)	76 / 642,545 (1.18)	123 / 1,705,746 (0.72)
**9**	812 / 3,846,584 (2.11)	622 / 1,601,391 (3.88)	58 / 663,816 (0.87)	132 / 1,581,377 (0.83)
**10**	654 / 3,404,284 (1.92)	484 / 1,413,928 (3.42)	55 / 625,664 (0.88)	115 / 1,364,690 (0.84)
**NHS England region**				
**East of England**	889 / 4,510,888 (1.97)	721 / 2,668,140 (2.7)	87 / 960,092 (0.91)	81 / 882,656 (0.92)
**London**	776 / 8,484,137 (0.91)	532 / 4,161,714 (1.28)	167 / 2,219,614 (0.75)	77 / 2,102,810 (0.37)
**Midlands**	1,869 / 8,929,204 (2.09)	1,369 / 3,570,726 (3.83)	82 / 702,650 (1.17)	418 / 4,655,829 (0.9)
**North East and Yorkshire**	1,509 / 7,519,277 (2.01)	829 / 1,438,286 (5.76)	94 / 489,701 (1.92)	586 / 5,591,290 (1.05)
**North West**	893 / 5,612,248 (1.59)	521 / 1,154,706 (4.51)	64 / 399,443 (1.6)	308 / 4,058,099 (0.76)
**South East**	1,526 / 6,307,276 (2.42)	1,263 / 3,056,679 (4.13)	193 / 1,760,200 (1.1)	70 / 1,490,397 (0.47)
**South West**	629 / 2,550,199 (2.47)	541 / 1,686,918 (3.21)	18 / 165,180 (1.09)	70 / 698,100 (1)
**Specimen date**				
**1 Nov–14 Nov**	1,795 / 16,126,616 (1.11)	1,119 / 5,544,758 (2.02)	23 / 509,274 (0.45)	653 / 10,072,584 (0.65)
**15 Nov–28 Nov**	1,457 / 8,609,193 (1.69)	1,057 / 2,918,120 (3.62)	32 / 609,924 (0.52)	368 / 5,081,148 (0.72)
**29 Nov–12 Dec**	1,510 / 5,932,273 (2.55)	1,085 / 2,179,776 (4.98)	133 / 1,315,496 (1.01)	292 / 2,437,001 (1.2)
**13 Dec–26 Dec**	1,838 / 7,905,330 (2.33)	1,363 / 4,087,120 (3.33)	281 / 2,470,546 (1.14)	194 / 1,347,664 (1.44)
**27 Dec–11 Jan**	1,491 / 5,339,816 (2.79)	1,152 / 3,007,394 (3.83)	236 / 1,791,640 (1.32)	103 / 540,782 (1.9)

536

medRxiv preprint doi: https://doi.org/10.1101/2021.02.01.21250959; this version posted February 3, 2021. The copyright holder for this preprint (which was not certified by peer review) is the author/funder, who has granted medRxiv a license to display the preprint in perpetuity.
It is made available under a CC-BY 4.0 International license .

Table S5. Hazard ratios for SGTF / VOC across models.

Model	Parameter	Hazard ratio (95% CI)	P value
**SGTF + lin age + lin IMD | NHSE + spec week**	sgtf	1.40 (1.23–1.59)	< 0.001
**SGTF + lin age + lin IMD | UTLA + spec week**	sgtf	1.36 (1.17–1.57)	< 0.001
**SGTF + lin age + lin IMD | LTLA + spec week**	sgtf	1.34 (1.15–1.56)	< 0.001
**SGTF + lin age + lin IMD | NHSE + spec date**	sgtf	1.40 (1.23–1.60)	< 0.001
**SGTF + lin age + lin IMD | UTLA + spec date**	sgtf	1.33 (1.13–1.57)	< 0.001
**SGTF + lin age + lin IMD | LTLA + spec date**	sgtf	1.30 (1.09–1.56)	0.003
**SGTF + spl age + spl IMD | NHSE + spec week**	sgtf	1.40 (1.23–1.59)	< 0.001
**SGTF + spl age + spl IMD | UTLA + spec week**	sgtf	1.36 (1.17–1.57)	< 0.001
**SGTF + spl age + spl IMD | LTLA + spec week**	sgtf	1.34 (1.15–1.56)	< 0.001
**SGTF + spl age + spl IMD | NHSE + spec date**	sgtf	1.40 (1.22–1.60)	< 0.001
**SGTF + spl age + spl IMD | UTLA + spec date**	sgtf	1.33 (1.13–1.57)	< 0.001
**SGTF + spl age + spl IMD | LTLA + spec date**	sgtf	1.31 (1.1–1.56)	0.003
**Censoring: SGTF_07 + lin age + lin IMD | LTLA + spec week**	sgtf	1.06 (0.81–1.38)	0.674
**Censoring: SGTF_14 + lin age + lin IMD | LTLA + spec week**	sgtf	1.19 (1.00–1.43)	0.052
**Censoring: SGTF_21 + lin age + lin IMD | LTLA + spec week**	sgtf	1.29 (1.10–1.51)	0.002
**Censoring: SGTF_28 + lin age + lin IMD | LTLA + spec week**	sgtf	1.34 (1.15–1.56)	< 0.001
**Censoring: SGTF_60 + lin age + lin IMD | LTLA + spec week**	sgtf	1.32 (1.14–1.53)	< 0.001
**Censoring: SGTF999 + lin age + lin IMD | LTLA + spec week**	sgtf	1.32 (1.14–1.52)	< 0.001
**Time-age interaction term | LTLA + spec date**	sgtf	1.30 (1.09–1.56)	0.003
**Time^2-age interaction term | LTLA + spec date**	sgtf	1.30 (1.09–1.55)	0.004
**Time-sex interaction term | LTLA + spec date**	sgtf	1.30 (1.09–1.56)	0.003

medRxiv preprint doi: https://doi.org/10.1101/2021.02.01.21250959; this version posted February 3, 2021. The copyright holder for this preprint (which was not certified by peer review) is the author/funder, who has granted medRxiv a license to display the preprint in perpetuity.
It is made available under a CC-BY 4.0 International license .

**Time^2-sex interaction term | LTLA + spec date**	sgtf	1.30 (1.09–1.56)	0.003
**Time-IMD interaction term | LTLA + spec date**	sgtf	1.30 (1.09–1.56)	0.003
**Time^2-IMD interaction term | LTLA + spec date**	sgtf	1.3 (1.09–1.55)	0.004
**Time-ethnicity interaction term | LTLA + spec date**	sgtf	1.31 (1.09–1.56)	0.003
**Time^2-ethnicity interaction term | LTLA + spec date**	sgtf	1.31 (1.09–1.56)	0.003
**Time-residence interaction term | LTLA + spec date**	sgtf	1.30 (1.09–1.56)	0.003
**Time^2-residence interaction term | LTLA + spec date**	sgtf	1.3 (1.09–1.56)	0.003
**Sensitivity: Sep onwards (SGTF + lin age + lin IMD | LTLA + spec date)**	sgtf	1.25 (1.05–1.48)	0.011
**Sensitivity: Oct onwards (SGTF + lin age + lin IMD | LTLA + spec date)**	sgtf	1.25 (1.05–1.48)	0.011
**Sensitivity: Nov onwards (SGTF + lin age + lin IMD | LTLA + spec date)**	sgtf	1.30 (1.09–1.56)	0.003
**Sensitivity: Dec onwards (SGTF + lin age + lin IMD | LTLA + spec date)**	sgtf	1.32 (1.09–1.61)	0.005
**Sensitivity: Prevalence cutoff (SGTF + lin age + lin IMD | LTLA + spec date)**	sgtf	1.32 (1.09–1.61)	0.005
**Sensitivity: no registration cutoff (SGTF + lin age + lin IMD | LTLA + spec date)**	sgtf	1.48 (1.28–1.70)	< 0.001
**Sensitivity: SGTF + lin age + lin IMD + NHSE * spec week**	sgtf	1.40 (1.23–1.60)	< 0.001
**p_voc + lin age + lin IMD | NHSE + spec week**	p_voc	1.48 (1.28–1.70)	< 0.001
**p_voc + lin age + lin IMD | UTLA + spec week**	p_voc	1.43 (1.22–1.67)	< 0.001
**p_voc + lin age + lin IMD | LTLA + spec week**	p_voc	1.4 (1.19–1.65)	< 0.001
**p_voc + lin age + lin IMD | NHSE + spec date**	p_voc	1.48 (1.29–1.71)	< 0.001
**p_voc + lin age + lin IMD | UTLA + spec date**	p_voc	1.38 (1.16–1.65)	< 0.001
**p_voc + lin age + lin IMD | LTLA + spec date**	p_voc	1.35 (1.11–1.63)	0.002
**p_voc + spl age + spl IMD | NHSE + spec week**	p_voc	1.48 (1.28–1.70)	< 0.001
**p_voc + spl age + spl IMD | UTLA + spec week**	p_voc	1.43 (1.22–1.67)	< 0.001
**p_voc + spl age + spl IMD | LTLA + spec week**	p_voc	1.41 (1.19–1.65)	< 0.001

medRxiv preprint doi: https://doi.org/10.1101/2021.02.01.21250959; this version posted February 3, 2021. The copyright holder for this preprint (which was not certified by peer review) is the author/funder, who has granted medRxiv a license to display the preprint in perpetuity.
It is made available under a CC-BY 4.0 International license .

**p_voc + spl age + spl IMD | NHSE + spec date**	p_voc	1.48 (1.28–1.71)	< 0.001
**p_voc + spl age + spl IMD | UTLA + spec date**	p_voc	1.38 (1.16–1.65)	< 0.001
**p_voc + spl age + spl IMD | LTLA + spec date**	p_voc	1.35 (1.12–1.64)	0.002

539

medRxiv preprint doi: https://doi.org/10.1101/2021.02.01.21250959; this version posted February 3, 2021. The copyright holder for this preprint (which was not certified by peer review) is the author/funder, who has granted medRxiv a license to display the preprint in perpetuity.
It is made available under a CC-BY 4.0 International license .

Supplementary Figures

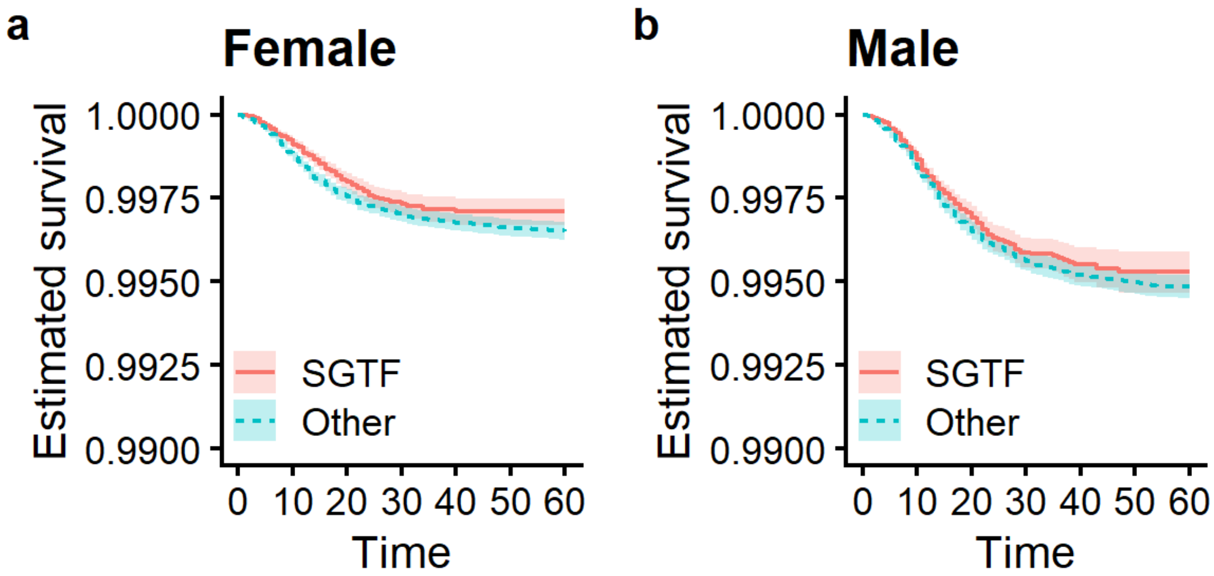

Fig. S1. Kaplan Meier plots of survival within 60 days of positive test for SGTF versus all other positive SARS-CoV-2 tests by sex.

medRxiv preprint doi: https://doi.org/10.1101/2021.02.01.21250959; this version posted February 3, 2021. The copyright holder for this preprint (which was not certified by peer review) is the author/funder, who has granted medRxiv a license to display the preprint in perpetuity.
It is made available under a CC-BY 4.0 International license .

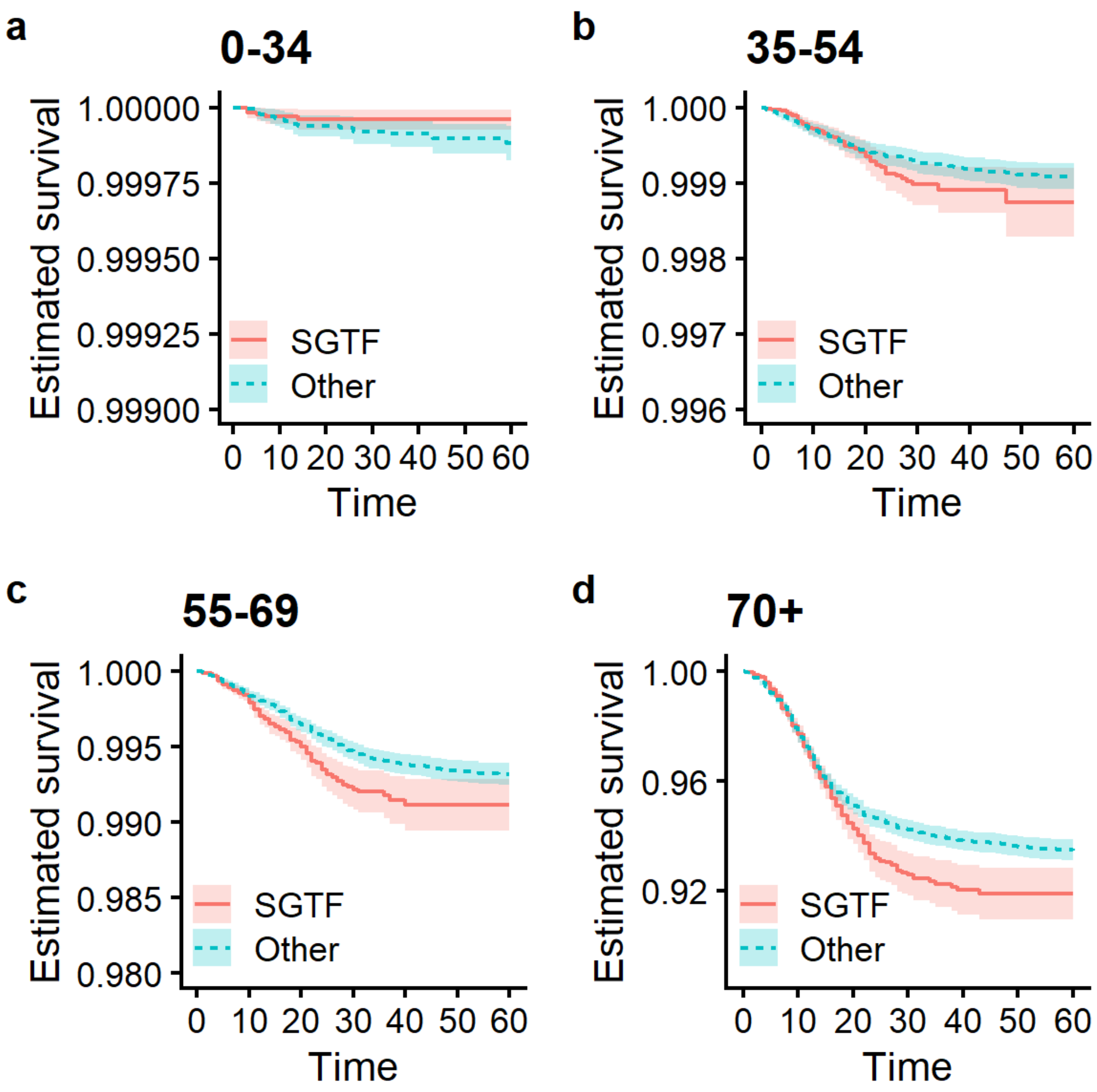

Fig. S2. Kaplan Meier plots of survival within 60 days of positive test for SGTF versus all other positive SARS-CoV-2 tests by age group. Note that the Y axis differs for each panel.

medRxiv preprint doi: https://doi.org/10.1101/2021.02.01.21250959; this version posted February 3, 2021. The copyright holder for this preprint
(which was not certified by peer review) is the author/funder, who has granted medRxiv a license to display the preprint in perpetuity.
It is made available under a CC-BY 4.0 International license .

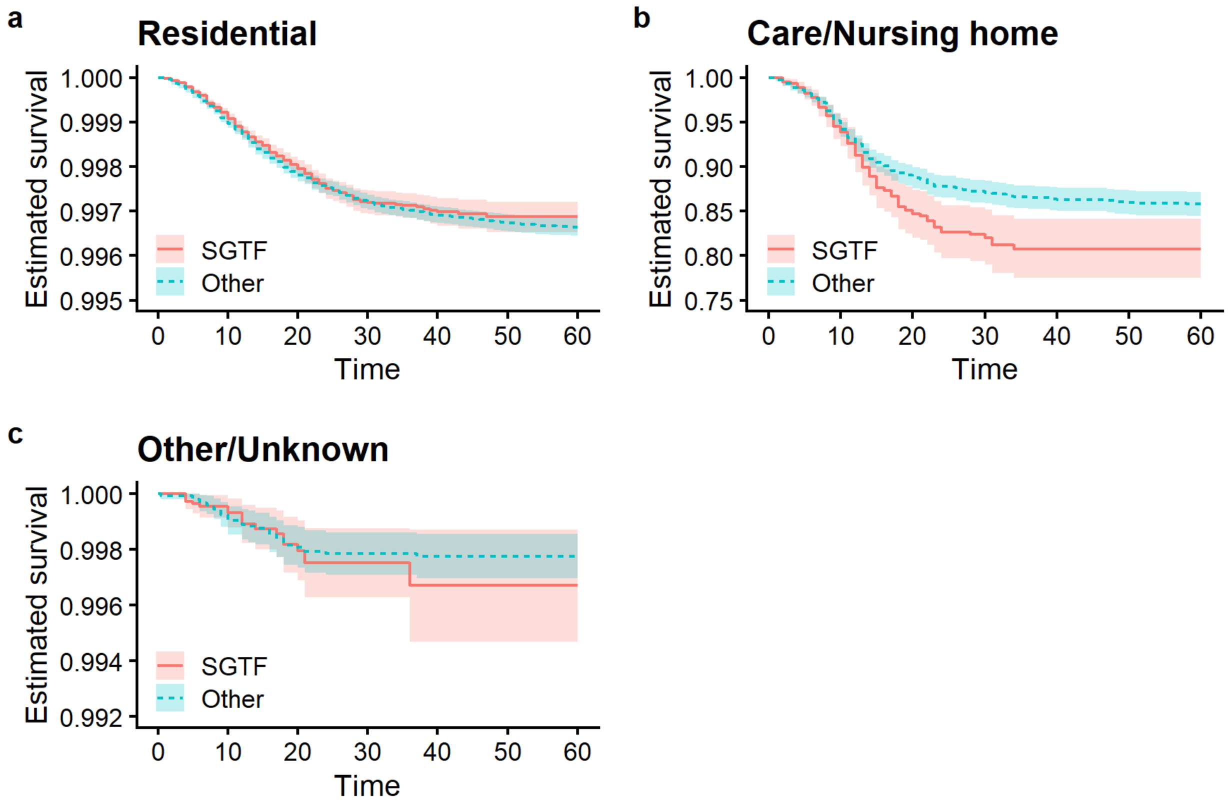

Fig. S3. Kaplan Meier plots of survival within 60 days of positive test for SGTF versus all other positive SARS-CoV-2 tests by residence. Note that the Y axis differs for each panel.

medRxiv preprint doi: https://doi.org/10.1101/2021.02.01.21250959; this version posted February 3, 2021. The copyright holder for this preprint (which was not certified by peer review) is the author/funder, who has granted medRxiv a license to display the preprint in perpetuity.
It is made available under a CC-BY 4.0 International license .

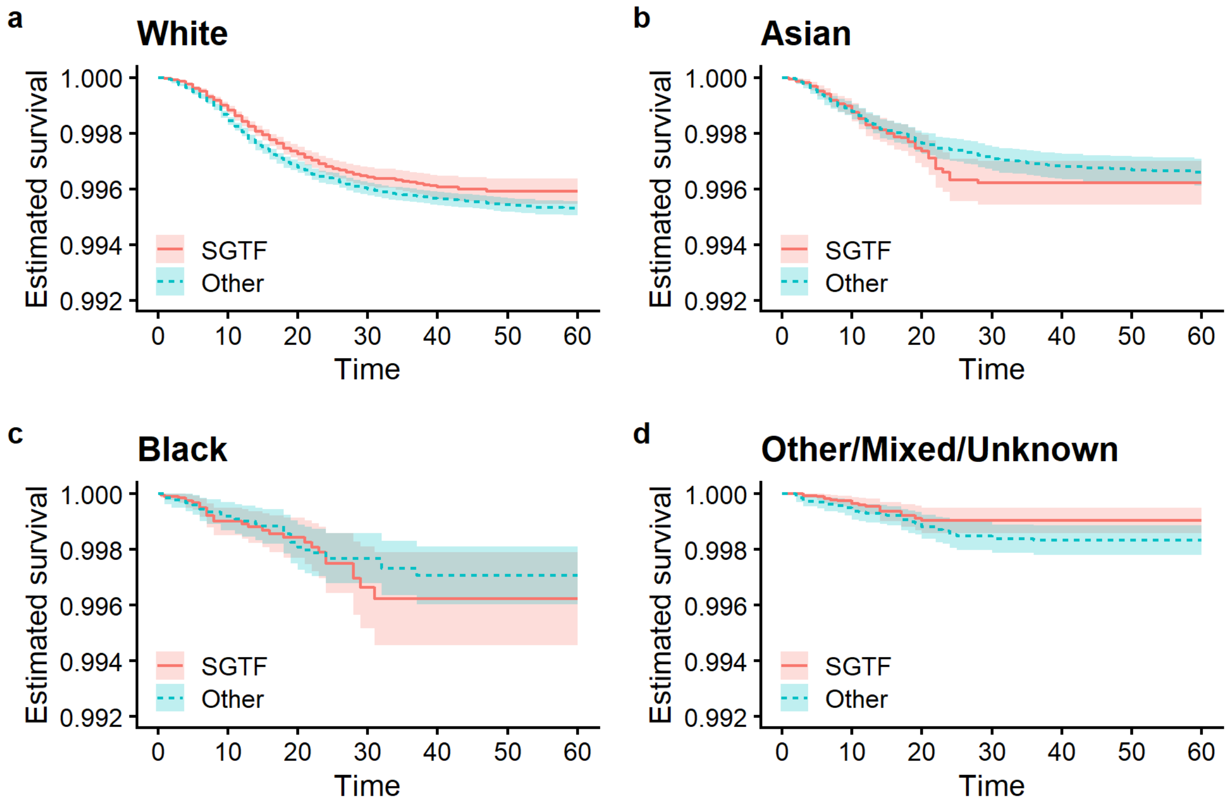

Fig. S4. Kaplan Meier plots of survival within 60 days of positive test for SGTF versus all other positive SARS-CoV-2 tests by Ethnicity.

medRxiv preprint doi: https://doi.org/10.1101/2021.02.01.21250959; this version posted February 3, 2021. The copyright holder for this preprint (which was not certified by peer review) is the author/funder, who has granted medRxiv a license to display the preprint in perpetuity.
It is made available under a CC-BY 4.0 International license .

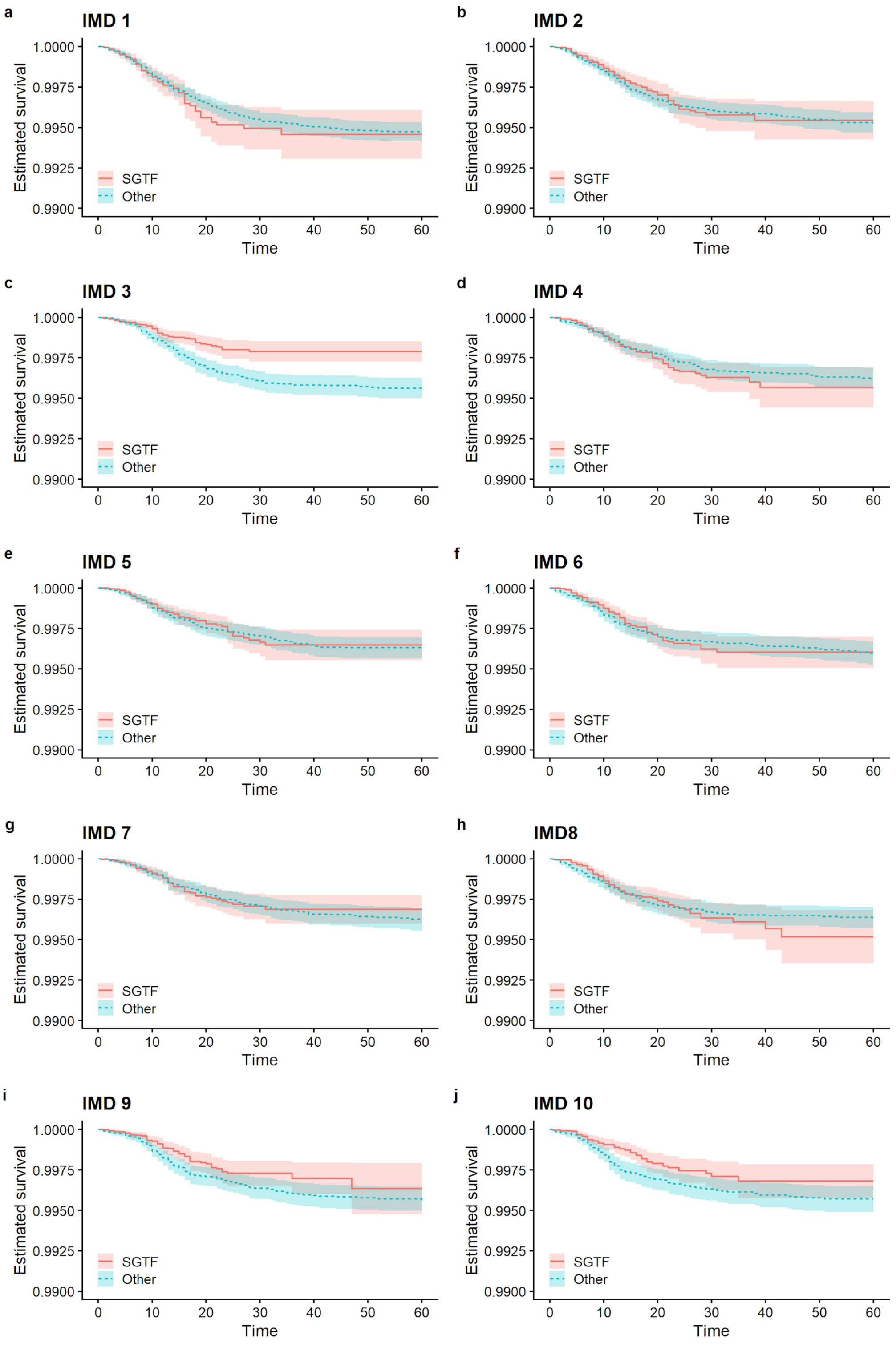

Fig. S5. Kaplan Meier plots of survival within 60 days of positive test for SGTF versus all other positive SARS-CoV-2 tests by Index of Multiple Deprivation decile (IMD).

medRxiv preprint doi: https://doi.org/10.1101/2021.02.01.21250959; this version posted February 3, 2021. The copyright holder for this preprint (which was not certified by peer review) is the author/funder, who has granted medRxiv a license to display the preprint in perpetuity.
It is made available under a CC-BY 4.0 International license .

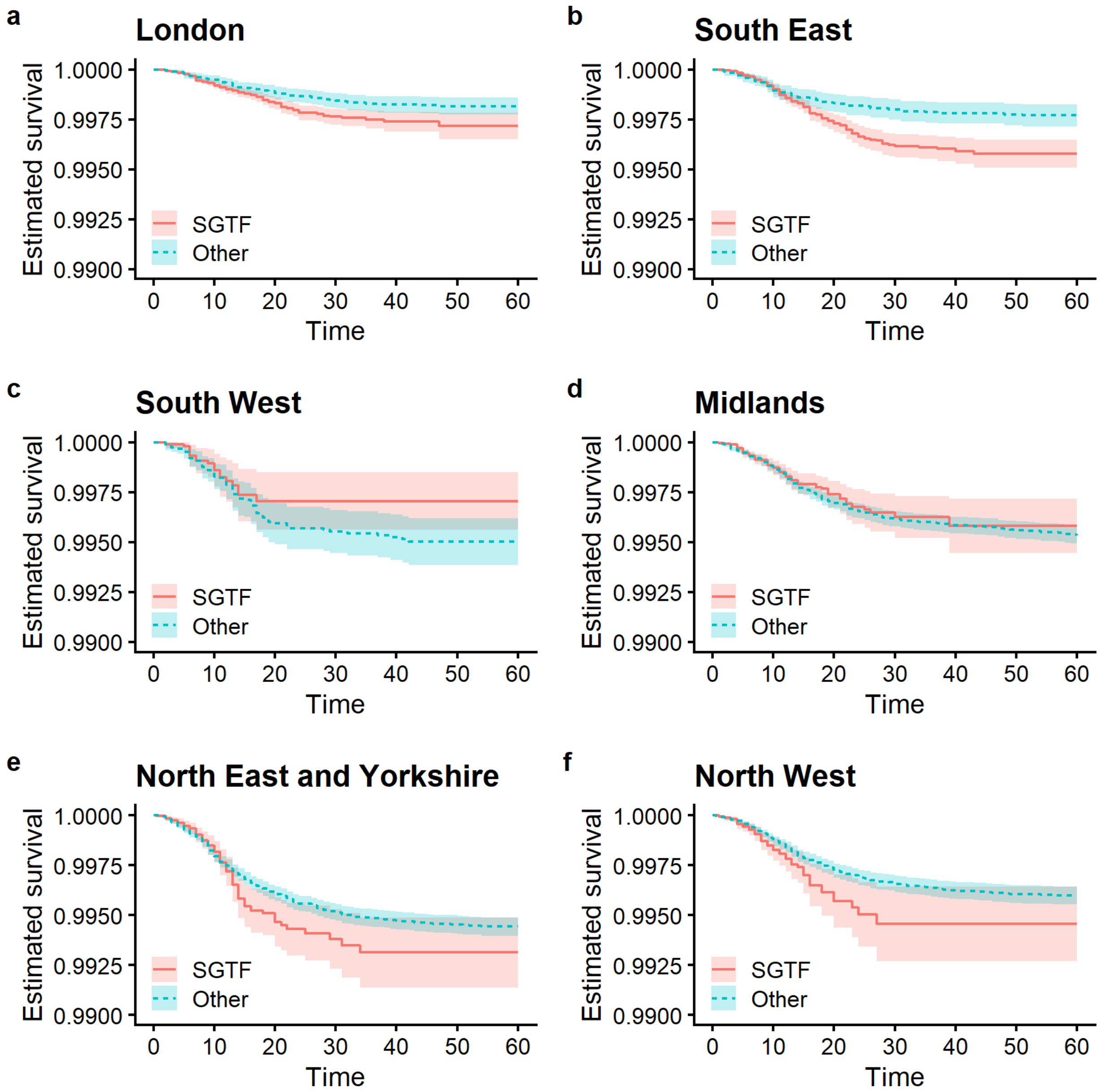

Fig. S6. Kaplan Meier plots of survival within 60 days of positive test for SGTF versus all other positive SARS-CoV-2 tests by NHS region.

medRxiv preprint doi: https://doi.org/10.1101/2021.02.01.21250959; this version posted February 3, 2021. The copyright holder for this preprint
(which was not certified by peer review) is the author/funder, who has granted medRxiv a license to display the preprint in perpetuity.
It is made available under a CC-BY 4.0 International license .

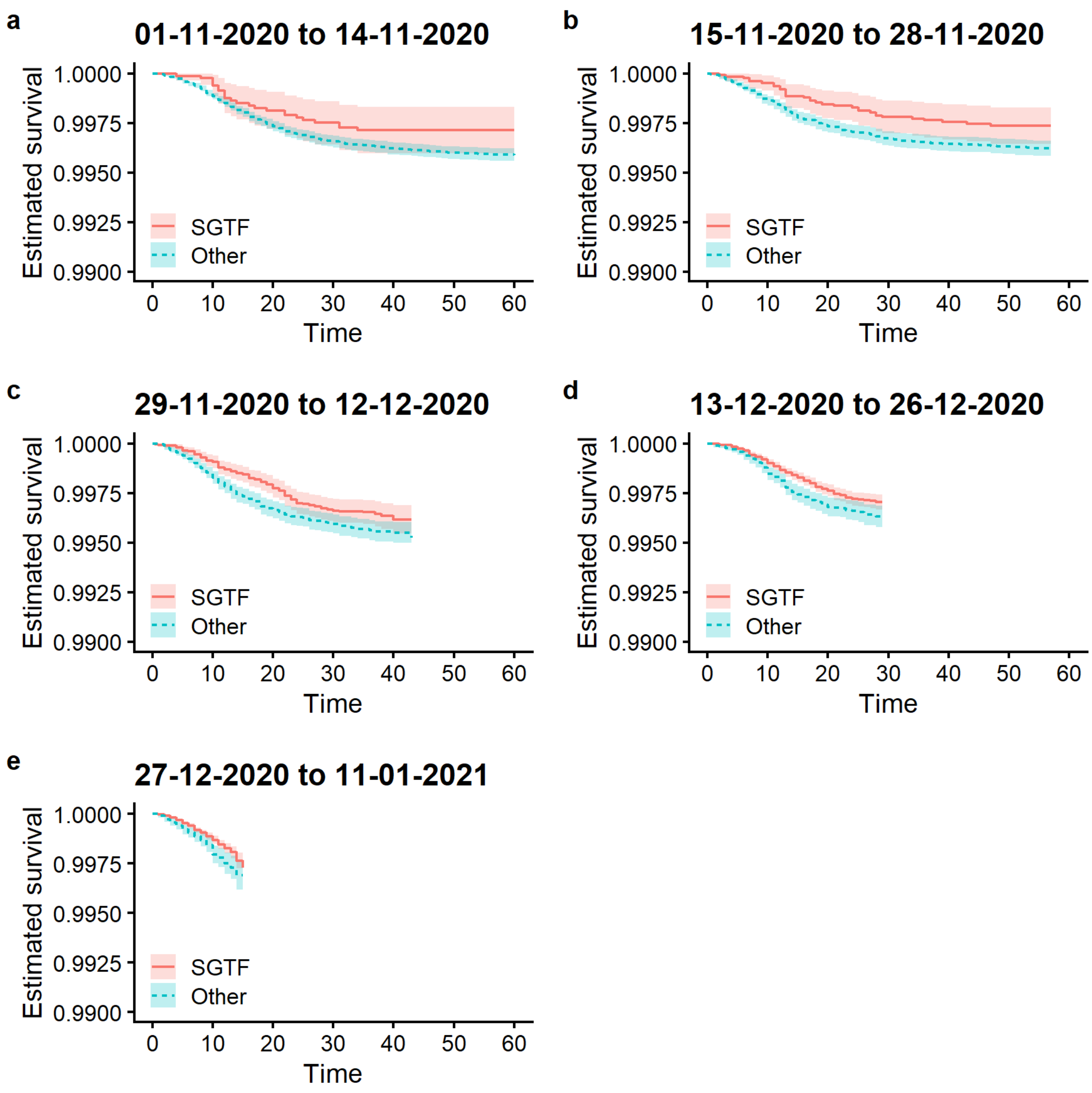

Fig. S7. Kaplan Meier plots of survival within 60 days of positive test for SGTF versus all other positive SARS-CoV-2 tests by specimen date.

medRxiv preprint doi: https://doi.org/10.1101/2021.02.01.21250959; this version posted February 3, 2021. The copyright holder for this preprint
(which was not certified by peer review) is the author/funder, who has granted medRxiv a license to display the preprint in perpetuity.
It is made available under a CC-BY 4.0 International license .

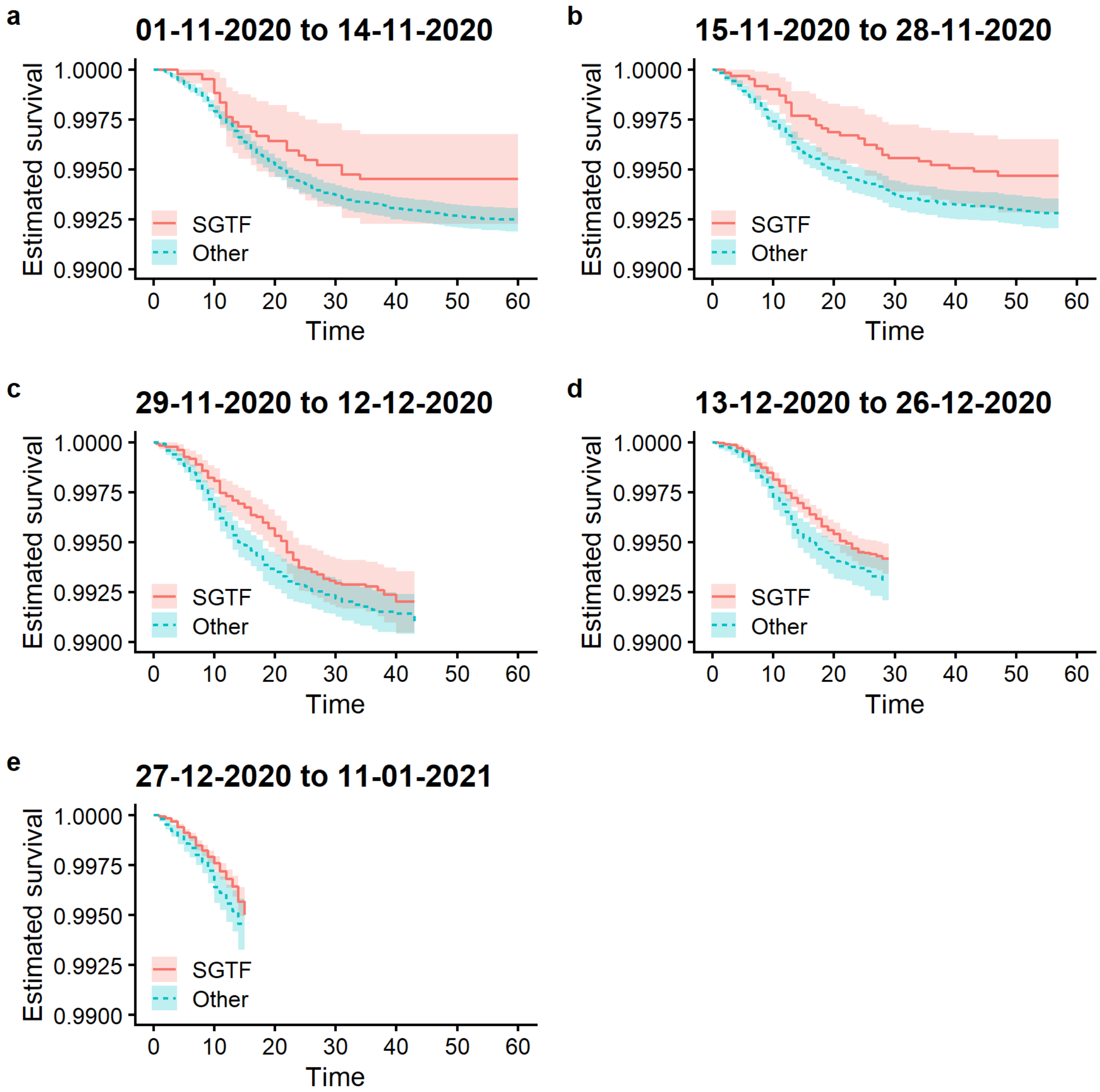

Fig. S8. Kaplan Meier plots of survival within 60 days of positive test for SGTF versus all other positive SARS-CoV-2 tests by specimen date for cases 35 years of age or older.

medRxiv preprint doi: https://doi.org/10.1101/2021.02.01.21250959; this version posted February 3, 2021. The copyright holder for this preprint (which was not certified by peer review) is the author/funder, who has granted medRxiv a license to display the preprint in perpetuity.
It is made available under a CC-BY 4.0 International license .

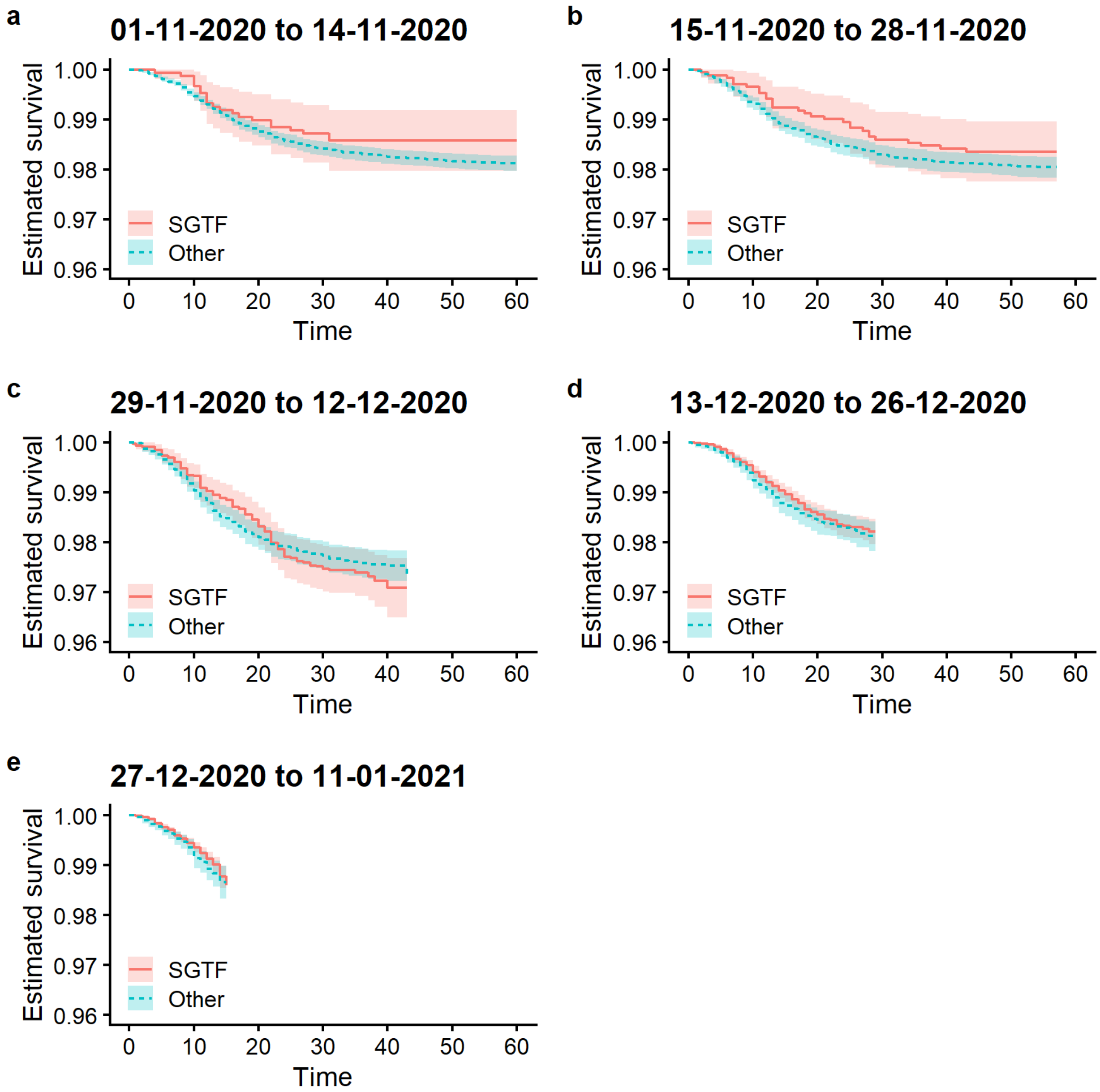

Fig. S9. Kaplan Meier plots of survival within 60 days of positive test for SGTF versus all other positive SARS-CoV-2 tests by specimen date for cases 55 years of age or older.

medRxiv preprint doi: https://doi.org/10.1101/2021.02.01.21250959; this version posted February 3, 2021. The copyright holder for this preprint (which was not certified by peer review) is the author/funder, who has granted medRxiv a license to display the preprint in perpetuity.
It is made available under a CC-BY 4.0 International license .

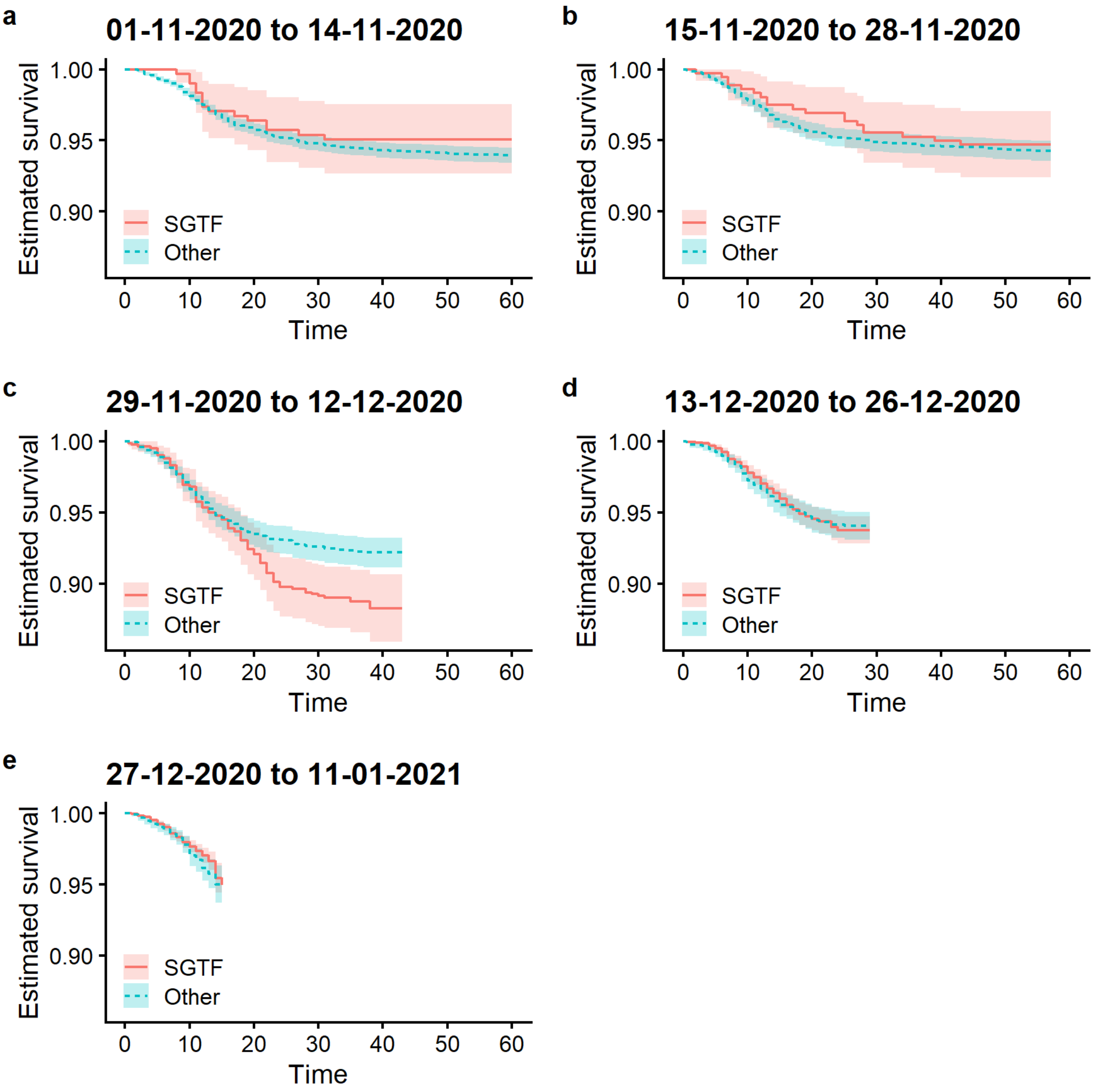

Fig. S10. Kaplan Meier plots of survival within 60 days of positive test for SGTF versus all other positive SARS-CoV-2 tests by specimen date for cases 70 years of age or older.

medRxiv preprint doi: https://doi.org/10.1101/2021.02.01.21250959; this version posted February 3, 2021. The copyright holder for this preprint (which was not certified by peer review) is the author/funder, who has granted medRxiv a license to display the preprint in perpetuity.
It is made available under a CC-BY 4.0 International license .

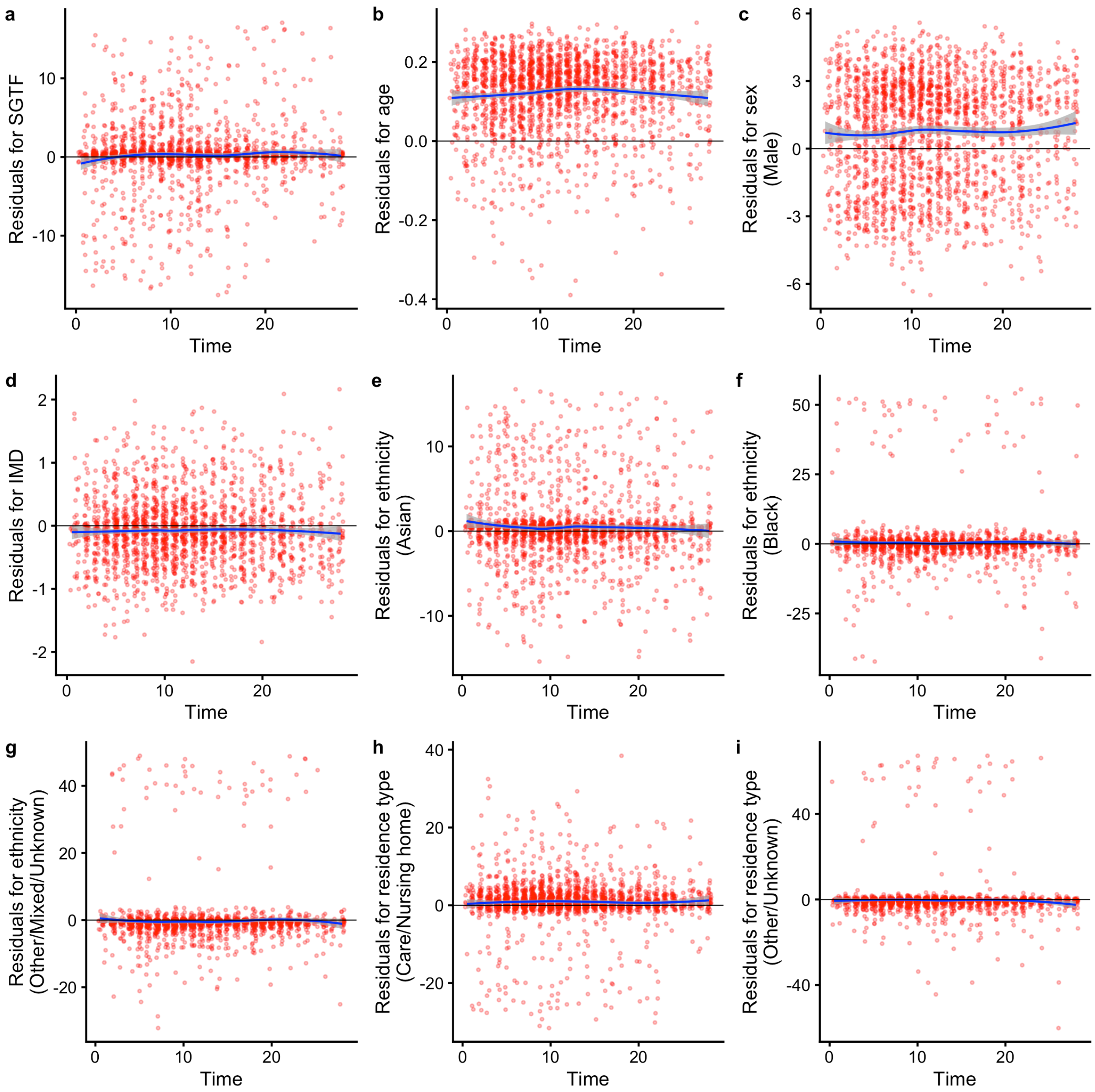

Fig. S11. Schoenfeld residuals for survival model by SGTF stratified by LTLA and specimen date. Model uses linear terms for age and IMD and a 28-day followup. Schoenfeld residual tests give *P* = 0.031 for SGTF; *P* = 0.425 for age; *P* = 0.170 for sex; *P* = 0.603 for IMD decile; *P* = 0.410 for ethnicity; *P* = 0.728 for residence type; and *P* = 0.244 globally.

medRxiv preprint doi: https://doi.org/10.1101/2021.02.01.21250959; this version posted February 3, 2021. The copyright holder for this preprint (which was not certified by peer review) is the author/funder, who has granted medRxiv a license to display the preprint in perpetuity.
It is made available under a CC-BY 4.0 International license .

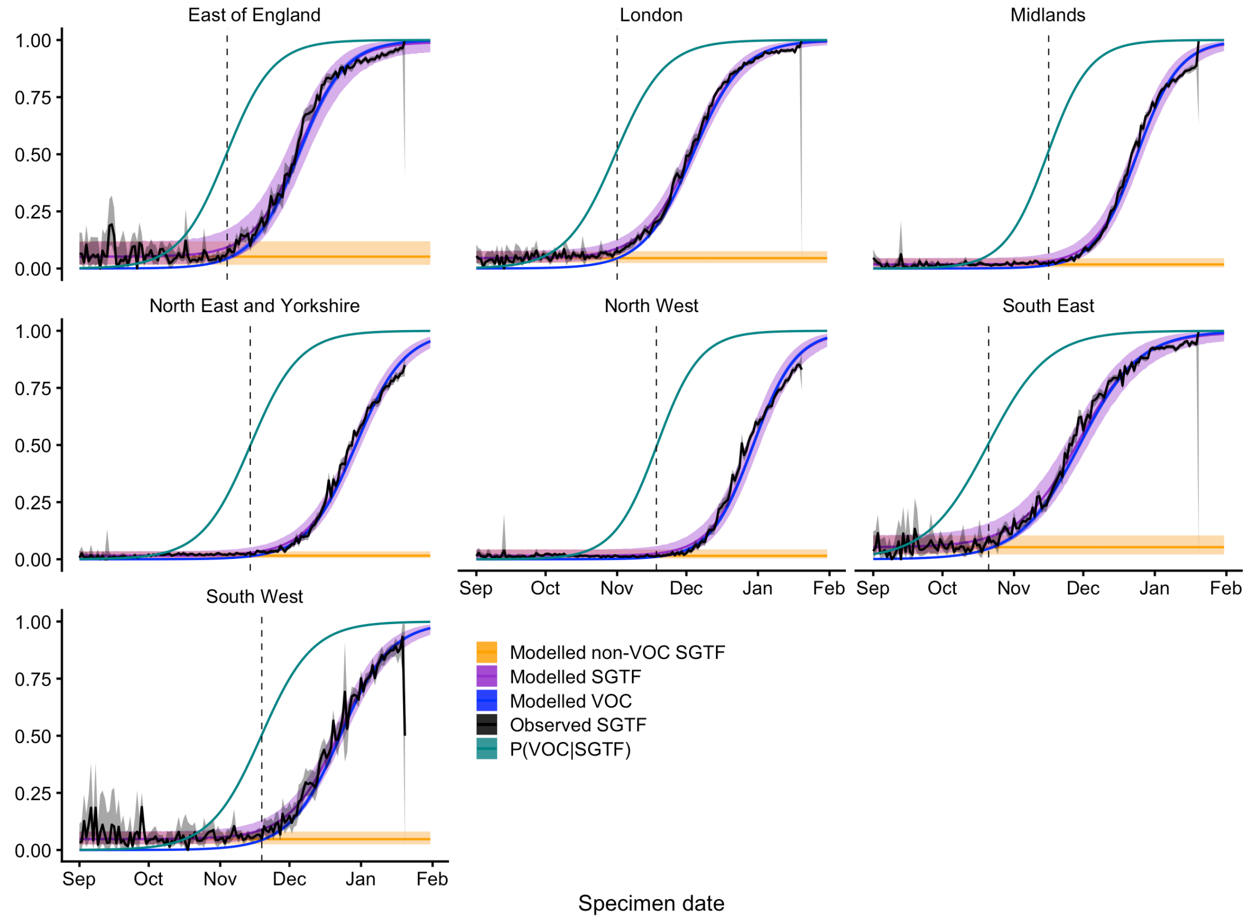

603
604 **Fig. S12. Misclassification model.** For each NHS England region, we fit a beta-binomial model
605 (purple, Modelled SGTF) to the observed SGTF frequencies among Pillar 2 tests (black,
606 Observed SGTF), which estimates a constant proportion of "false positive" SGTF samples
607 among non-VOC 202012/01 specimens (orange, Modelled non-VOC SGTF) and a logistically
608 growing proportion of VOC 202012/01 specimens over time (blue, Modelled VOC). This allows
609 us to model the conditional probability that a specimen with SGTF represents VOC 202012/01
610 (teal, P(VOC|SGTF)). For our misclassification survival analysis, p_{VOC} = 0 for non-SGTF
611 specimens and p_{VOC} = P(VOC|SGTF) for SGTF specimens.

medRxiv preprint doi: https://doi.org/10.1101/2021.02.01.21250959; this version posted February 3, 2021. The copyright holder for this preprint (which was not certified by peer review) is the author/funder, who has granted medRxiv a license to display the preprint in perpetuity.
It is made available under a CC-BY 4.0 International license .

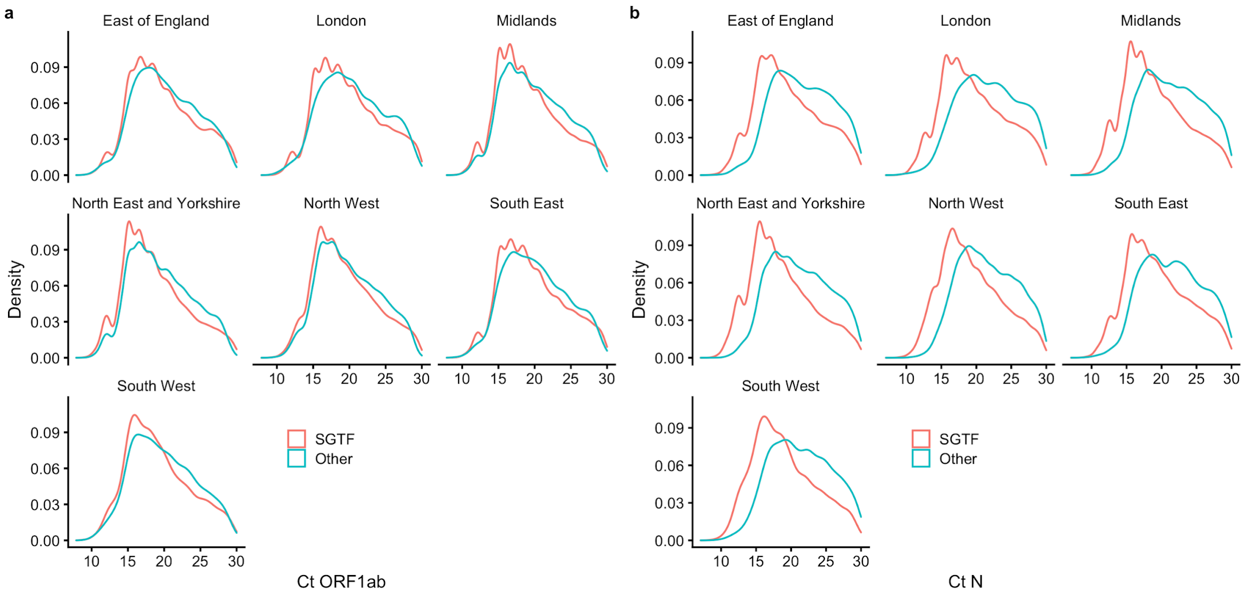

Fig. S13. Ct values for SGTF versus other. The distribution of Ct values for (**a**) ORF1ab and (**b**) N gene targets among specimens collected between 1–11 January 2021.
